# Thymidine Phosphodiester Chemiluminescent Probe for Sensitive and Selective Detection of Ectonucleotide Pyrophosphatase 1

**DOI:** 10.1021/acs.bioconjchem.4c00454

**Published:** 2025-01-09

**Authors:** Omri Shelef, Sara Gutkin, Molhm Nassir, Anne Krinsky, Ronit Satchi-Fainaro, Phil S. Baran, Doron Shabat

**Affiliations:** † School of Chemistry, Raymond and Beverly Sackler Faculty of Exact Sciences, Tel-Aviv University, Tel Aviv 69978, Israel; ‡ Department of Chemistry, Scripps Research, La Jolla, California 92037, United States; § Department of Physiology and Pharmacology, Faculty of Medical and Health Sciences, Tel Aviv University, Tel Aviv 6997801, Israel; ∥ Sagol School of Neuroscience, Tel Aviv University, Tel Aviv 6997801, Israel; ⊥ Center for Nanoscience and Nanotechnology, Tel Aviv University, Tel Aviv 6997801, Israel; # Cancer Biology Research Center, Tel Aviv University, Tel Aviv 6997801, Israel

## Abstract

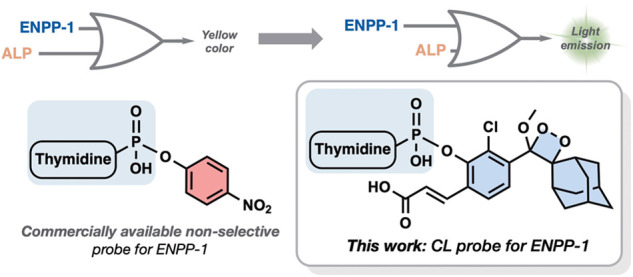

ENPP-1 is a transmembrane enzyme involved in nucleotide metabolism, and its overexpression is associated with various cancers, making it a potential therapeutic target and biomarker for early tumor diagnosis. Current detection methods for ENPP-1 utilize a colorimetric probe, **TMP-*****p*****NP**, which has significant limitations in sensitivity. Here, we present probe **CL-ENPP-1**, the first nucleic acid-based chemiluminescent probe designed for rapid and highly sensitive detection of ENPP-1 activity. The design of probe **CL-ENPP-1** features a phenoxy-adamantyl-1,2-dioxetane luminophore linked to thymidine via a phosphodiesteric bond. Upon cleavage of the enzymatic substrate by ENPP-1, the probe undergoes an efficient chemiexcitation process to emit a green photon. Probe **CL-ENPP-1** demonstrates an exceptional signal-to-noise ratio of 15000 and a limit of detection value approximately 4500-fold lower than the widely used colorimetric probe **TMP-*****p*****NP**. A comparison of **TMP-*****p*****NP** activation by ENPP-1 versus alkaline phosphatase (ALP) reveals a complete lack of selectivity. Removal of the self-immolative spacer from probe **CL-ENPP-1** resulted in a new chemiluminescent probe, **CL-ENPP-2**, with an 18.4-fold increase in selectivity for ENPP-1 over ALP. The ability of probe **CL-ENPP-2** to detect ENPP-1 activity in mammalian cells was assessed using the human breast cancer cell line MDA-MB-231. This probe demonstrated a 19.5-fold improvement in the signal-to-noise ratio, highlighting its superior ability to detect ENPP-1 activity in a biological sample. As far as we know, to date, **CL-ENPP-1** and **CL-ENPP-2** are the most sensitive probes for the detection of ENPP-1 catalytic activity. We anticipate that our new chemiluminescent probes will be valuable for various applications requiring ENPP-1 detection, including enzyme inhibitor-based drug discovery assays. The insights gained from our probe design principles could advance the development of more selective probes for ENPP-1 and contribute to future innovations in chemiluminescence research.

## Introduction

Ectonucleotide pyrophosphatase/phosphodiesterase 1 (ENPP-1) is a transmembrane glycoprotein that converts extracellular nucleotide triphosphates, such as ATP, into nucleotide monophosphates, AMP and inorganic pyrophosphate.^1^ This enzyme is a key member of the NPP family, which includes ENPP-1 – ENPP-7, ecto-nucleoside tripho diphosphohydrolases, and ecto-5′-nucleotidase.^2^ These NPPs regulate extracellular nucleotide levels, which are crucial signaling molecules in nearly all cell types, tissues, and organs.^1,2^ The overexpression of ENPP-1 has been linked to various cancers, making it a potential therapeutic target and a biomarker for early tumor diagnosis.^3,4^

Due to its importance, a commercially available colorimetric probe for the detection of ENPP-1 was developed about 50 years ago.^5^ Since then, *p*-nitrophenyl 5′-thymidine monophosphate (**TMP-*****p*****NP**) has been the most commonly used substrate for monitoring the enzymatic activity of ENPP-1, largely due to its straightforward procedure, which enables high-throughput screening of compound libraries by simply monitoring the formation of yellow-colored *p*-nitrophenolate. Although colorimetric measurements are routinely performed, they suffer from low sensitivity and typically require prolonged incubation periods (18–24 h).^6^ Since fluorescent methods generally provide improved detection sensitivities compared to colorimetric methods, a new fluorescent probe for ENPP-1 was developed five years ago.^7,8^ This probe consists of the fluorophore Tokyo Green, which is linked to adenosine through a phosphodiester bond. In the presence of ENPP-1, the phosphodiester bond is cleaved, releasing Tokyo Green, which can then be excited at a maximum absorbance peak of 455 nm, resulting in a fluorescent signal with a maximum emission peak of 515 nm.

Since both absorbance and fluorescence operation mode requires an external light source, phenomena such as light scattering and autofluorescence can occur, leading to increased background signal and reduced sensitivity.^9^ In contrast, the operation mode of chemiluminescence generates light through a chemical reaction without the need for an external light source. This inherent advantage eliminates background interference and thus results in superior detection sensitivity.^10−12^

Due to their exceptional sensitivity, chemiluminescent probes have emerged as versatile and powerful tools for diagnostic and imaging applications.^13^ Among these chemiluminescent probes, phenoxy-dioxetanes have attracted remarkable attention. By linking the phenol to a specifically tailored responsive group, chemiexcitation can be controlled and initiated in the presence of a specific enzyme or a bioanalyte of interest.^14^ Although the discovery of phenoxy-dioxetanes held great promise, their applications were limited due to poor light emission efficiency in aqueous solutions. To overcome this limitation, in 2017, our group discovered that incorporating an acrylate substituent at the *ortho* position of a phenoxy-adamantyl-1,2-dioxetane prevents water-mediated quenching of the excited intermediate, leading to an enhancement in light-emission intensity of up to 3000-fold.^15^ This pivotal discovery enabled researchers, for the first time, to use chemiluminescent probes in aqueous solutions without the need for additives, significantly expanding their potential applications in biological studies. These new dioxetane probes have been widely employed in bioimaging, immunoassays, and real-time monitoring of cellular events both *in vitro* and *in vivo*.^16−27^ Herein, we report the first nucleic acid-dioxetane chemiluminescent probe designed for selective and highly sensitive detection of ENPP-1.

## Results and Discussion

**Figure 1:**
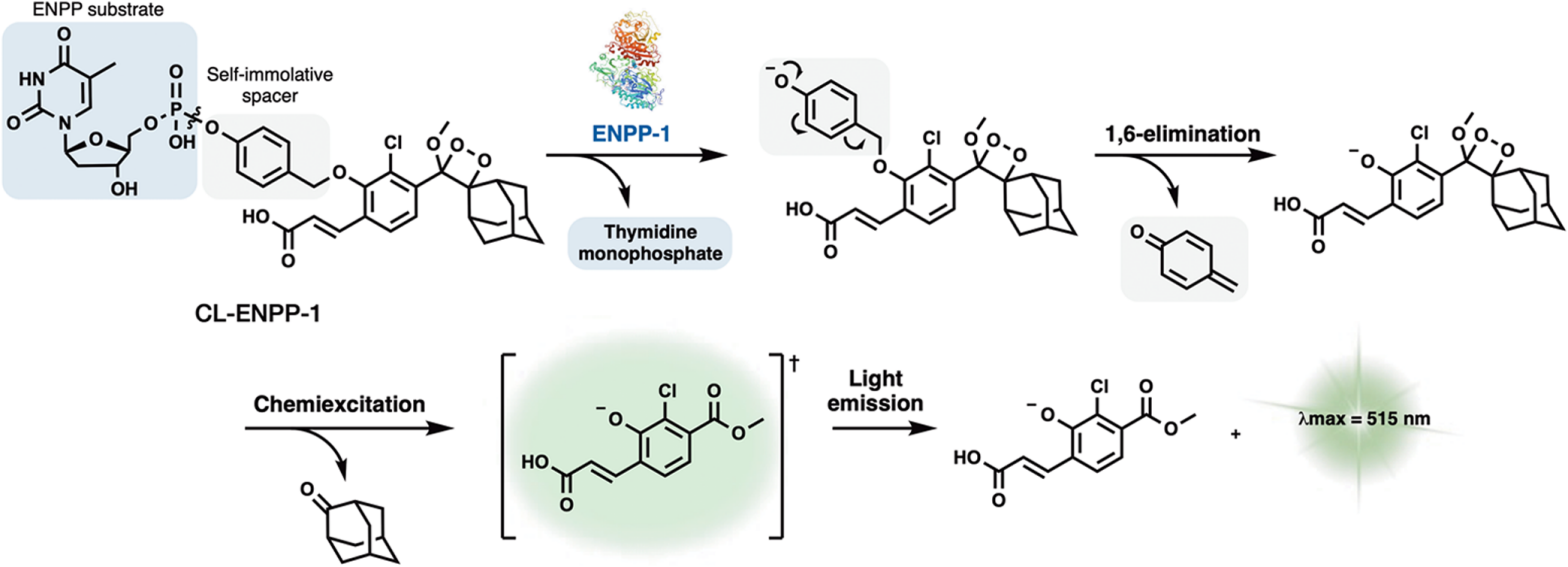
Chemiexcitation disassembly pathway of probe **CL-ENPP-1** upon reaction with ENPP-1.

The molecular structure of probe **CL-ENPP-1** and its chemiluminescence activation pathway are shown in Figure 1. The probe is composed of the ENPP-1 substrate, thymidine-monophosphate, conjugated through a short self-immolative linker to an adamantyl-phenoxy-1,2-dioxetane luminophore with an *ortho* acrylic acid substituent. Upon enzymatic cleavage of the phosphodiesteric bond by ENPP-1, a spontaneous 1,6-elimination of the spacer occurs, yielding a phenolate intermediate. This phenolate then undergoes a chemiexcitation process, forming an excited benzoate species that decays to its ground state through the emission of a green photon.

**Figure 2:**
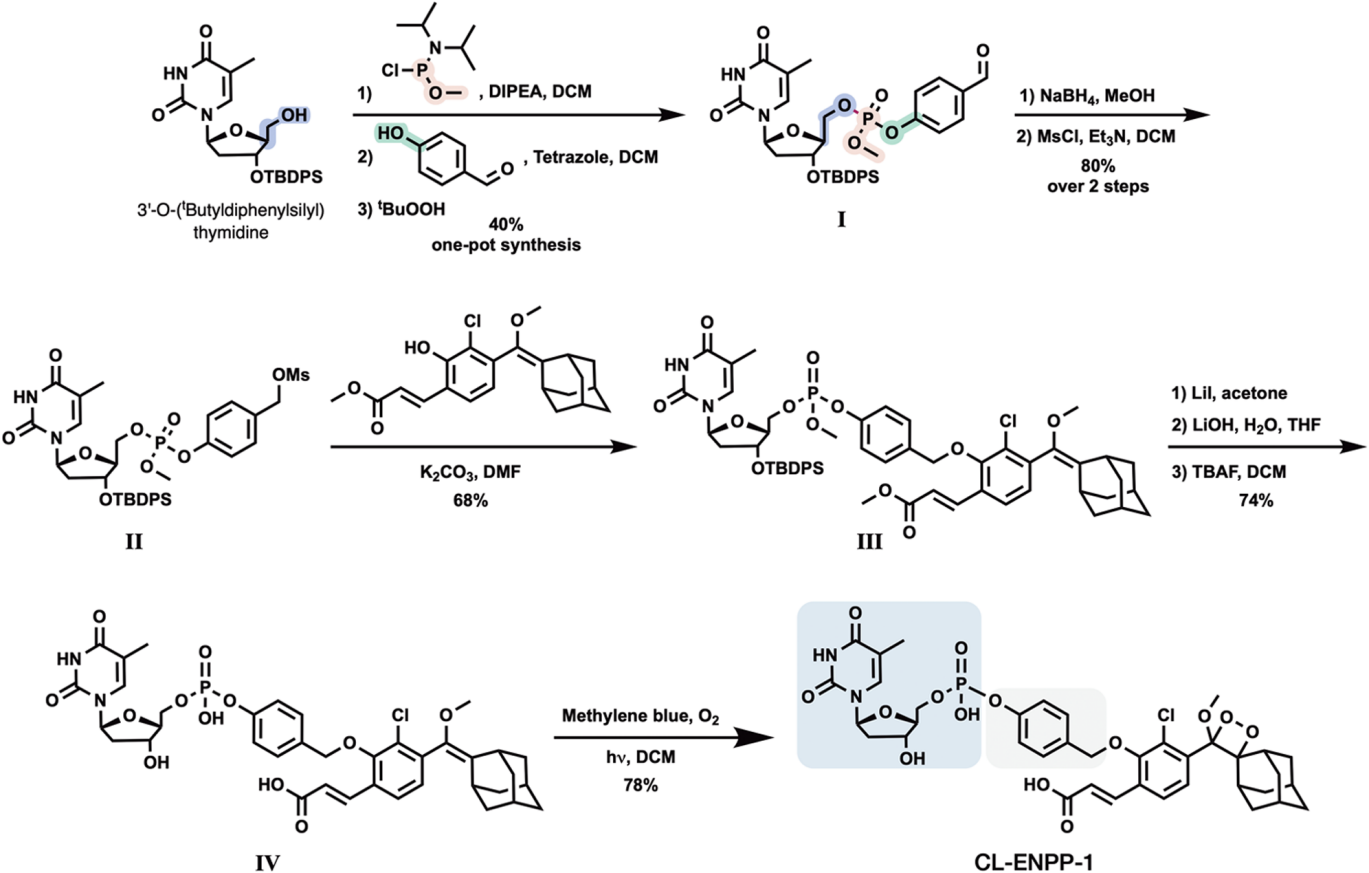
Synthetic pathway used for the preparation of probe **CL-ENPP-1**.

The synthesis of probe **CL-ENPP-1** was achieved as described in Figure 2. 3′-O-(*tert-*Butyldiphenylsilyl)-thymidine was conjugated to 4-hydroxybenzaldehyde using classic phosphoroamidate chemistry. The resulting aldehyde (**I**) was reduced with sodium borohydride to form a benzyl alcohol, which was then treated with methane sulfonyl chloride in the presence of triethylamine to produce benzyl mesylate (**II**). Nucleophilic substitution of benzyl mesylate (**II**) with the previously synthesized phenol enol ether^28^ yielded enol ether (**III**). The three protecting groups of enolether (**III**) were removed in the following steps: First, the methyl phosphotriester was demethylated with lithium iodide to form a phosphodiester; next, the methyl acrylate group was hydrolyzed using lithium hydroxide and water; and finally, the *tert*-butyldiphenylsilyl group was removed with tetra-butylammonium fluoride. The resulting deprotected enolether (**IV**) was then oxidized by singlet oxygen to produce the final probe, **CL-ENPP-1**. It should be noted, that the order of protecting group removal is of significant importance due to the relatively high reactivity of the phosphotriester group. This group is prone to rapid hydrolysis under aqueous basic conditions and may undergo an undesired intramolecular cyclization if the 3′ hydroxy group of the deoxyribose is not protected.

**Figure 3:**
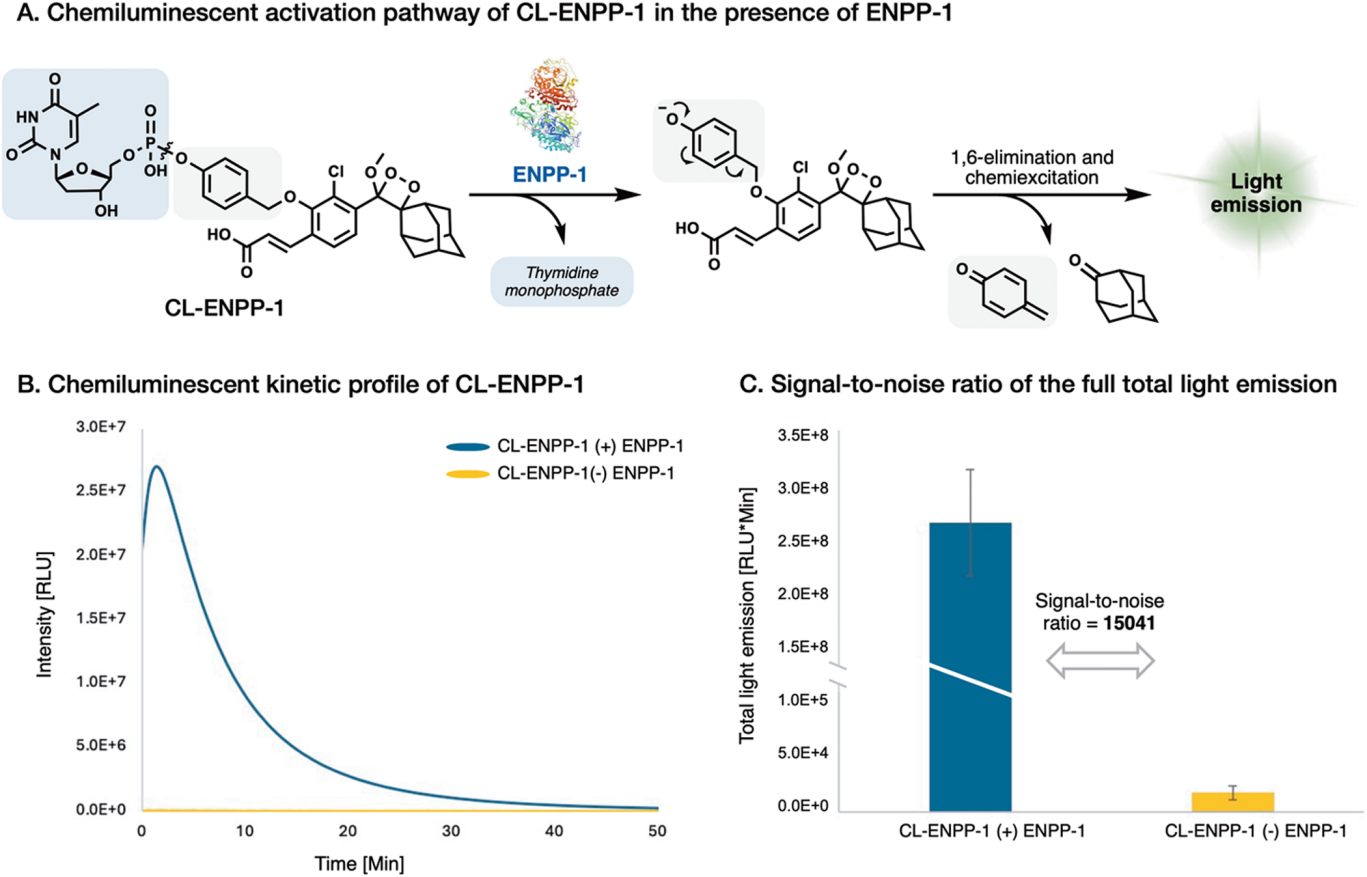
(A) Chemiluminescent activation pathway (B) Chemiluminescent kinetic profile and (C) total light emission of probe **CL-ENPP-1** [10 μM], in PBS pH 7.4, 1% DMSO, at 37 °C with or without Recombinant Human ENPP-1 [0.1 μg/mL].

Initially, we sought to evaluate the light-emission turn-on response and the chemiluminescent kinetic profile of probe **CL-ENPP-1** in the presence of ENPP-1 (Figure 3A). Probe **CL-ENPP-1** was incubated in an aqueous buffer (PBS, pH 7.4) with or without commercially available recombinant human ENPP-1. The chemiluminescent light emission profile and the total emitted light are presented in Figures 3B and 3C, respectively. In the presence of ENPP-1, probe **CL-ENPP-1** displays a typical chemiluminescent kinetic profile, that begins with a rapid increase in light emission intensity, followed by a gradual decay of the signal over 60 min. Remarkably, the total light-emission signal measured for probe **CL-ENPP-1** in the presence of ENPP-1 was about 15000-fold greater than that observed in the absence of the enzyme. This result represents a significantly high S/N, even for a chemiluminescent probe. The exceptional S/N is likely due to the high hydrolytic stability of the phosphodiester bond, which minimizes background signal, in combination with an excellent substrate compatibility of the probe with its target enzyme, ENPP-1.

**Figure 4:**
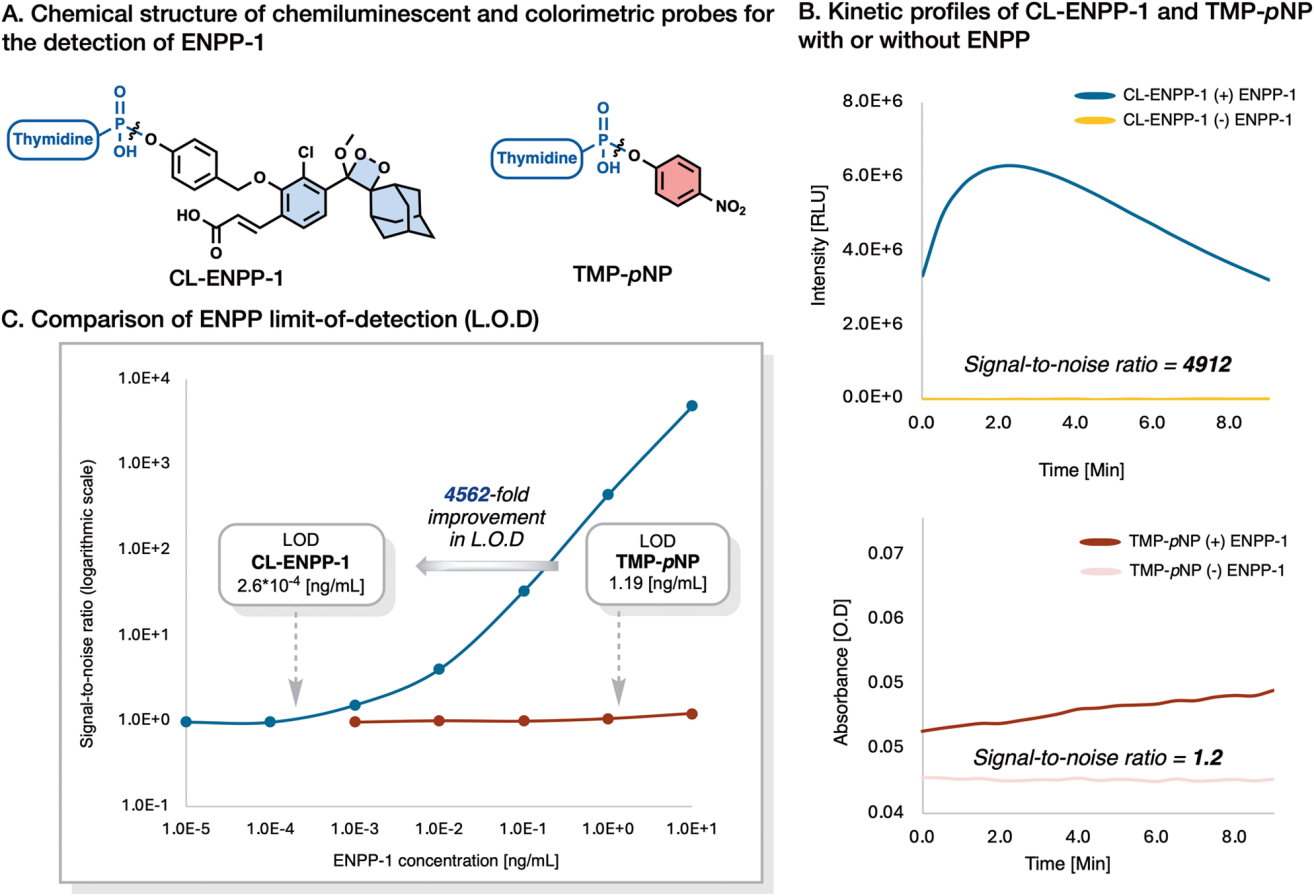
Comparison between the sensitivity of the chemiluminescent probe **CL-ENPP-1** and the commercially available colorimetric probe **TMP-*****p*****NP**. (A) Chemical structure, (B) kinetic profile, and (C) L.O.D of probe **CL-ENPP-1** [30 μM] and **TMP-*****p*****NP** [300 μM] in PBS pH 7.4, 3% DMSO, 37 °C with or without Recombinant Human ENPP-1 [0.1 μg/mL - 1 pg/mL].

We then aimed to compare the detection sensitivity of probe **CL-ENPP-1** for ENPP-1 activity with that of the commercially available colorimetric probe **TMP-*****p*****NP** (Figure 4A). The S/N ratios of both probes were determined in the presence and absence of ENPP-1. Probe **CL-ENPP-1** achieved a superior S/N ratio of 4912, which is approximately 4000-fold higher than the S/N ratio obtained for probe **TMP-*****p*****NP**, S/N value of 1.2 (Figure 4B).

We next conducted a comparative evaluation of the limit-of-detection (LOD) values of probe **CL-ENPP-1** and **TMP-*****p*****NP** in the presence of ENPP-1 (Figure 4C and supporting figures S1 and S2). Probe **CL-ENPP-1** exhibited an LOD value which was 4562-fold greater than that of **TMP-*****p*****NP**. These results demonstrate the superior ENPP-1 detection sensitivity of the chemiluminescent probe **CL-ENPP-1** compared to the commonly used colorimetric probe **TMP-*****p*****NP**.

The remarkable sensitivity of probe **CL-ENPP-1** compared to probe **TMP-*****p*****NP** encouraged us to further evaluate its selectivity for ENPP-1. The alkaline phosphatase (ALP) family is another group of enzymes that shares some functional overlap with NPPs and is abundantly expressed in various cell types, tissues, and organs.^1,6^ Therefore, to preliminarily assess whether these probes can selectively detect ENPP-1 in a cell-based assay containing various types of NPPs and ALPs, we compared the activation of the two probes in the presence of both ENPP-1 and ALP.

**Figure 5:**
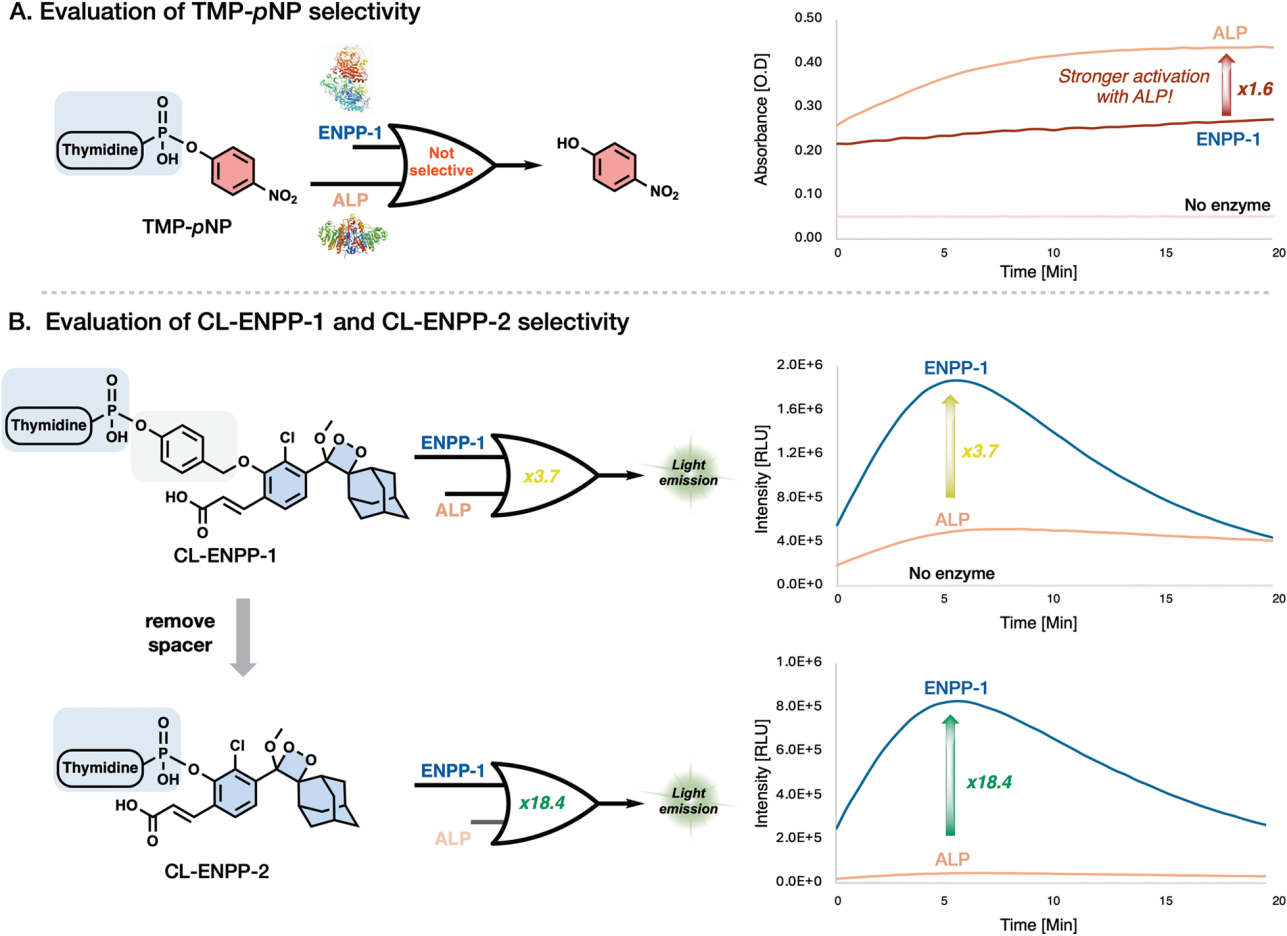
Evaluation of the selectivity for ENPP-1 over ALP. (A) Colorimetric kinetic profile of probe **TMP-*****p*****NP** [300 μM] and (B) chemiluminescent kinetic profile of probe **CL-ENPP-1** and probe **CL-ENPP-2** [10 μM] in PBS pH 7.4, 1% DMSO, 37 °C with or without Recombinant Human ENPP-1 [0.1 μg/mL] or Alkaline phosphatase [0.075U/mL].

Probe **CL-ENPP-1** and probe **TMP-*****p*****NP** were incubated with standard concentrations of commercially available ENPP-1 or ALP (from bovine intestinal mucosa). Their colorimetric or chemiluminescent light emission kinetic profiles are presented in Figure 5. Surprisingly, the commonly used probe **TMP-*****p*****NP** exhibited a 1.6-fold greater activation signal in the presence of ALP compared to ENPP-1 (Figure 5A). These results indicate that **TMP-*****p*****NP** suffers from a lack of selectivity for ENPP-1 and therefore, is not ideal for ENPP-1 detection in a cell-based assay. Conducting a similar measurement with probe **CL-ENPP-1** using the same enzymatic concentrations yielded improved selectivity. Unlike **TMP-*****p*****NP**, probe **CL-ENPP** demonstrated a stronger activation signal by ENPP-1, with a 3.7-fold increase in comparison to its activation by ALP (Figure 5B, Up).

A possible explanation for the different selectivity profiles of probe **TMP-*****p*****NP** and probe **CL-ENPP-1** might be the increased steric hindrance introduced by the acrylate substituent in the chemiluminescent probe. Previous studies have shown that a self-immolative spacer can improve enzymatic activity by adding length to the molecule, thereby reducing steric hindrance near the enzymatic substrate. Since probe **CL-ENPP-1** contains a self-immolative spacer that reduces steric hindrance compared to an equivalent probe without the spacer, we synthesized probe **CL-ENPP-2**, which shares a similar chemical structure with CL-ENPP-1 but lacks the self-immolative spacer. Indeed, probe **CL-ENPP-2** demonstrated a significant improvement in selectivity, showing an 18.4-fold stronger activation signal in the presence of ENPP-1 compared to ALP ((Figure 5B, Down).

These results show that improved selectivity for ENPP-1 over ALP can be achieved through chemical manipulation in the distance between the enzymatic substrate and the reporter unit. The improved selectivity obtained by probe **CL-ENPP-2** enables its use in applications requiring selective detection of ENPP-1 over ALP. However, further evaluation of its activity in the presence of other NPP family members, as well as cell-based inhibition assays are still required to establish the precise selectivity profile of probe **CL-ENPP-2**.

**Figure 6:**
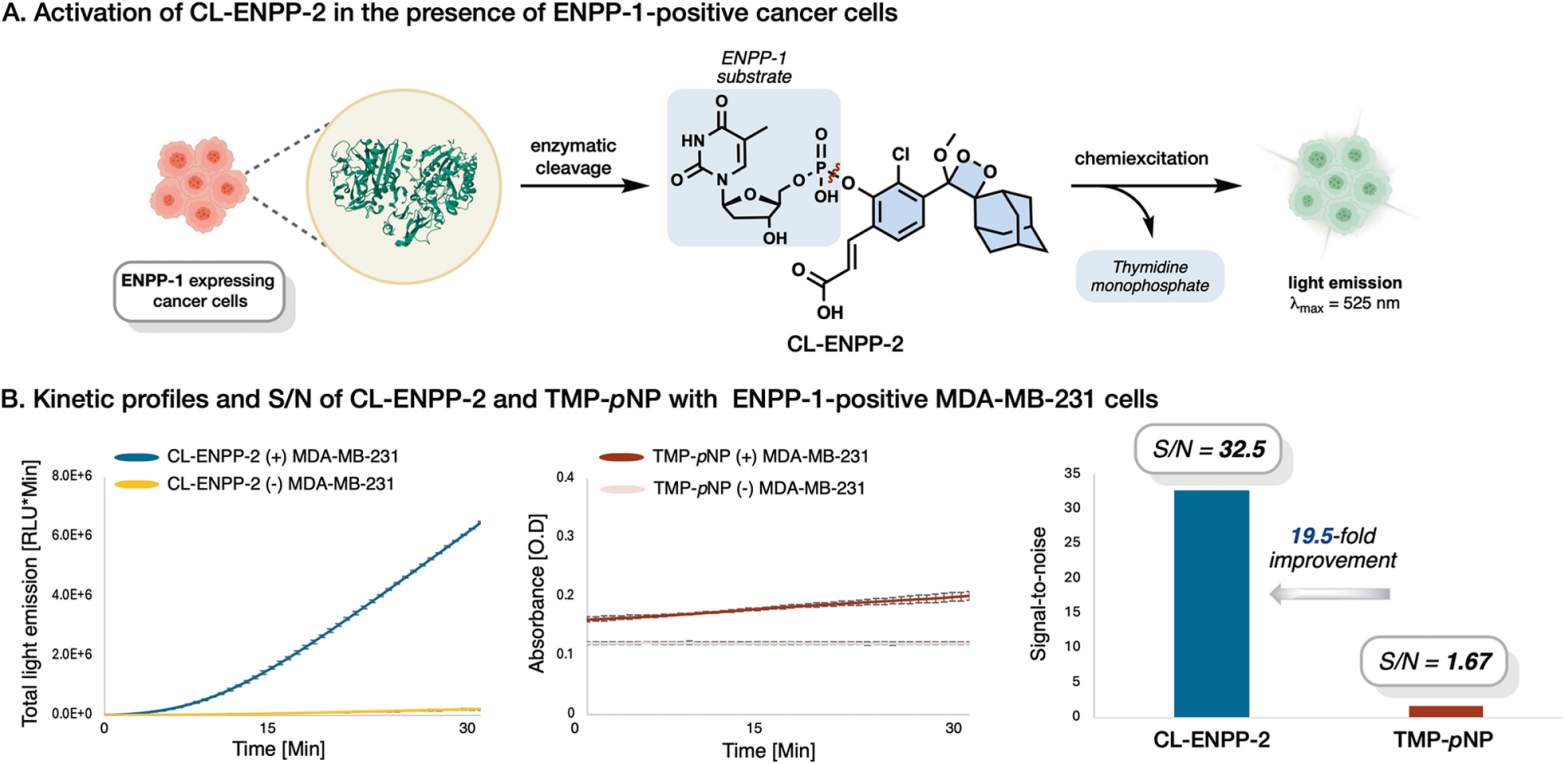
Comparison of **CL-ENPP-2** and **TMP-pNP** detection sensitivity for ENPP-1 in mammalian cells. (A) Chemiluminescent total light emission kinetic profile (left), colorimetric kinetic profile (middle), and signal-to-noise ratio comparison (right) of probes **CL-ENPP-2** [10 μM] and **TMP-pNP** [300 μM] in the presence or absence of MDA-MB-231 cells, in PBS pH 7.4, 0.1% DMSO, at 37 °C. For detailed procedures, refer to the experimental protocols section in the Supporting Information.

The exceptional detection sensitivity and improved selectivity of probe **CL-ENPP-2** toward ENPP-1 activity prompted us to evaluate its applicability for detecting enzymatic activity in mammalian cells. These measurements were conducted to assess the probe’s anti-interference capability and the reliability of its detection outcomes, in comparison to the commonly used **TMP-*****p*****NP** (Figure 6). Human breast cancer cells (MDA-MB-231) were selected for this comparison due to previous reports of ENPP-1 overexpression in this cell line.^7^ Probes **CL-ENPP-2** and **TMP-*****p*****NP** were incubated with MDA-MB-231 cells and the obtained optical signals were monitored for 30 min. The chemiluminescent signal generated by probe **CL-ENPP-2** in the presence of MDA-MB-231 cells was about 32-fold higher than the signal produced in the control (PBS, pH 7.4). On the other hand, the colorimetric signal produced by probe **TMP-*****p*****NP** in the presence of MDA-MB-231 cells was only 1.67-fold higher than the control. Overall probes **CL-ENPP-2** presented about 20-fold higher S/N compared to probe **TMP-*****p*****NP**. These results effectively demonstrate the superior ability of our chemiluminescent dioxetane probe over a colorimetric probe to detect ENPP-1 activity in MDA-MB-231 tumoral cells.

Since the discovery that *ortho* acrylate-substituted phenoxy-1,2-dioxetane luminophores significantly enhance light-emission intensity in water, numerous chemiluminescent probes have been developed for the detection of various enzymes and bioanalytes. Although most of these probes achieve high sensitivity with large S/N ratios, probe **CL-ENPP-1** is among the most sensitive chemiluminescent probes ever reported. This exceptional sensitivity is due to two key factors: the high hydrolytic stability of the phosphodiesteric bond, which minimizes background signal, and the excellent compatibility of the substrate with its target enzyme, ENPP-1. These two factors enable probe **CL-ENPP-1** to detect ENPP-1 with a notably high S/N ratio of 15000.

Currently, the commercial colorimetric probe **TMP-*****p*****NP** is the most commonly used probe for detecting ENPP-1 activity, due to its straightforward procedure and the limited availability of alternative detection methods. However, probe **CL-ENPP-1** offers several advantages over **TMP-*****p*****NP**: it provides rapid results within minutes, unlike colorimetric methods that can take up to 24 h; it has high chemical stability, enabling an easy-to-handle and straightforward procedure; and most importantly, it demonstrates superior detection capabilities with a 4500-fold lower LOD value. We, therefore, envision that probe **CL-ENPP-1** could replace **TMP-*****p*****NP**, facilitating highly sensitive detection of ENPP-1 activity that could enable high-throughput enzymatic screening of compound libraries. As far as we know, to date, **CL-ENPP-1** is the most sensitive probe for the detection of ENPP-1 enzymatic activity.

Further evaluation of probe **CL-ENPP-1** and probe **TMP-*****p*****NP** selectivity revealed unexpected phenomena. **TMP-*****p*****NP** exhibited a higher detection signal toward ALP activity compared to ENPP-1, while probe **CL-ENPP-1** demonstrated slightly better selectivity, with a 3.7-fold higher detection signal toward activity of ENPP-1 compared to ALP. These results suggest that while these probes are suitable for enzymatic assays, such as inhibitor screening, they are not suitable for more complex applications such as cell-based assays, where both ALP and ENPP-1 are presented. To improve the selectivity that requires ENPP-1 detection, we developed probe **CL-ENPP-2**. Shortening the space between the enzymatic substrate and the chemiluminescent reporter, by removal of the self-immolative spacer, has resulted in an increase of the steric hindrance near the enzymatic substrate, leading to an 18.4-fold higher S/N with ENPP-1 compared to ALP. Nevertheless, further evaluation of the probe **CL-ENPP-2** selectivity in the presence of other NPP family members is still required.

## Conclusion

In summary, we have developed the first nucleic acid–based chemiluminescent probe for the direct detection of ENPP-1 activity. The activation mechanism of probe **CL-ENPP-1** is based on hydrolytic cleavage of the substrate thymidine monophosphate, followed by a rapid chemiexcitation process that results in light emission. Probe **CL-ENPP-1** demonstrates a significant turn-on response with a notably high S/N ratio of 15000, which is relatively high compared to equivalent chemiluminescent probes. When compared with the commercially available **TMP-*****p*****NP**, probe **CL-ENPP-1** shows superior detection sensitivity, with an approximately 4500-fold improvement in the LOD value. The selectivity of the probes was evaluated against the widely abundant ALP. Probe **TMP-*****p*****NP** showed almost no selectivity, probe **CL-ENPP-1** exhibited moderate selectivity of 3.7-fold. Probe **CL-ENPP-2** obtained by reducing the molecular distance between the enzymatic substrate and the chemiluminescent reporter, showed an improved selectivity of 18.4-fold. These findings suggest that, unlike **TMP-*****p*****NP**, probe **CL-ENPP-2** is suitable for applications requiring selective detection of ENPP-1 over ALP. Finally, we demonstrated the superior detection sensitivity of probe **CL-ENPP-2** for ENPP-1 activity in tumor cells compared to probe **TMP-pNP**. Probe **CL-ENPP-2** exhibited a 19.5-fold improvement in the signal-to-noise (S/N) ratio over **TMP-pNP**. We anticipate that our new chemiluminescent probes will be valuable for various applications requiring ENPP-1 detection, including enzyme inhibitor-based drug discovery assays. The insights gained from our probe design principles could advance the development of more selective probes for ENPP-1 and contribute to future innovations in chemiluminescence research.

## Supporting Information Available

Synthetic procedures and characterization of all compounds, including ^1^H NMR, ^13^C NMR, HPLC, and MS, as well as experimental protocols and supplementary figures (PDF)
