# Supplementary material for: Thymidine Phosphodiester Chemiluminescent Probe for Sensitive and Selective Detection of Ectonucleotide Pyrophosphatase 1
Source: Bioconjug Chem. 2025 Jan 9;36(2):152–9. doi: 10.1021/acs.bioconjchem.4c00454 (PMC11843602; doi:10.1021/acs.bioconjchem.4c00454)
Supplement: Supplementary file 1 — bc4c00454_si_001.pdf [file bc4c00454_si_001.pdf]

Supporting Information

Thymidine Phosphodiester Chemiluminescent Probe for Sensitive and Selective Detection of Ectonucleotide Pyrophosphatase 1

Omri Shelef[a], Sara Gutkin[a], Molhm Nassir[b], Anne Krinsky[c], Ronit Satchi-Fainaro[c,d,e,f], Phil S. Baran[b] and Doron Shabat[a]*

[a]School of Chemistry, Raymond and Beverly Sackler Faculty of Exact Sciences, Tel-Aviv University, Tel Aviv 69978 Israel.

[b]Department of Chemistry, Scripps Research, La Jolla, CA 92037, USA.

[c]Department of Physiology and Pharmacology, Faculty of Medical and Health Sciences, Tel Aviv University, Tel Aviv 6997801, Israel.

[d]Sagol School of Neuroscience, Tel Aviv University, Tel Aviv 6997801, Israel.

[e]Center for Nanoscience and Nanotechnology, Tel Aviv University, Tel Aviv 6997801, Israel.

[f]Cancer Biology Research Center, Tel Aviv University, Tel Aviv 6997801, Israel.

*__Corresponding Authors:__

Doron Shabat, Email: chdoron@tauex.tau.ac.il

Table of Contents

General methods 3

Synthetic procedures and characterization of CL-ENPP-1 and CL-ENPP-2 4

General synthetic schemes 4

Synthesis of probe CL-ENPP-1 5

Synthesis of probe CL-ENPP-2 11

Experimental protocols 14

Chemiluminescent measurements of probes CL-ENPP-1 and CL-ENP-2 14

ENPP-1 Limit-of-detection measurements 14

Evaluation of the selectivity of the probe towards ENPP-1 compared to alkaline phosphatase 15

Detection of ENPP-1 activity in cancer cells in vitro 15

Supplementary Figures 16

HPLC and Mass Spectra 18

References 20

General methods

All reactions requiring anhydrous conditions were performed under an Argon atmosphere. All reactions were carried out at room temperature unless stated otherwise. Chemicals and solvents were either A.R. grade or purified by standard techniques. Thin-layer chromatography (TLC): silica gel plates Merck 60 F254: compounds were visualized by irradiation with UV light. Column chromatography (FC): silica gel Merck 60 (particle size 0.040-0.063 mm), eluent given in parentheses. Reverse-phase high-pressure liquid chromatography (RP-HPLC): C18 5u, 250x4.6mm, eluent given in parentheses. Preparative RP-HPLC: C18 5u, 250x21mm, eluent given in parentheses. ^{1}H-NMR spectra were measured using Bruker Avance operated at 400MHz. ^{13}C-NMR spectra were measured using Bruker Avance operated at 100 MHz. Chemical shifts were reported in ppm on the δ scale relative to a residual solvent ($CDCl_3$: δ = 7.26 for ^{1}H-NMR and 77.16 for ^{13}C-NMR, DMSO-d_6: δ = 2.50 for ^{1}H-NMR and 39.52 for ^{13}C-NMR and MeOD: δ = 3.31 for ^{1}H-NMR and 49.00 for ^{13}C-NMR). Mass spectra were measured on Waters Xevo TQD. Chemiluminescence was recorded on Molecular Devices Spectramax iD3. All general reagents, including salts and solvents, were purchased from Sigma-Aldrich. Light irradiation for photochemical reactions: LED PAR38 lamp (19W, 3000K).

Abbreviations

ACN- Acetonitrile, **$CHCl_3$**- Chloroform, **DCM**- Dichloromethane, **DIPEA**- N,N-Diisopropylethylamine, **DMF**- N,N'-Dimethylformamide, **DMAP**- 4-Dimethylaminopyridine, **EtOAc**- Ethyl acetate, **Et_3N**- Triethylamine, **Hex**- Hexanes, **K_2CO_3**- Potassium carbonate, **LI**- Lithium iodide, **LiOH**- Lithium hydroxide, **MB**- Methylene blue, **MeOH**- Methanol, **MsCl**- Mesyl chloride, **NH_4Cl**- Ammonium chloride, **$NaHCO_3$**- Sodium bicarbonate, **Na_2SO_4**- Sodium Sulfate, **TBAF**- Tetra-n-butylammonium fluoride, **TBDP**- Tert-butyldiphenylsilyl, **THF**- Tetrahydrofuran, **^{t}BuOOH**- Tert-Butyl hydroperoxide.

Synthetic procedures and characterization of CL-ENPP-1 and CL-ENPP-2

General synthetic schemes

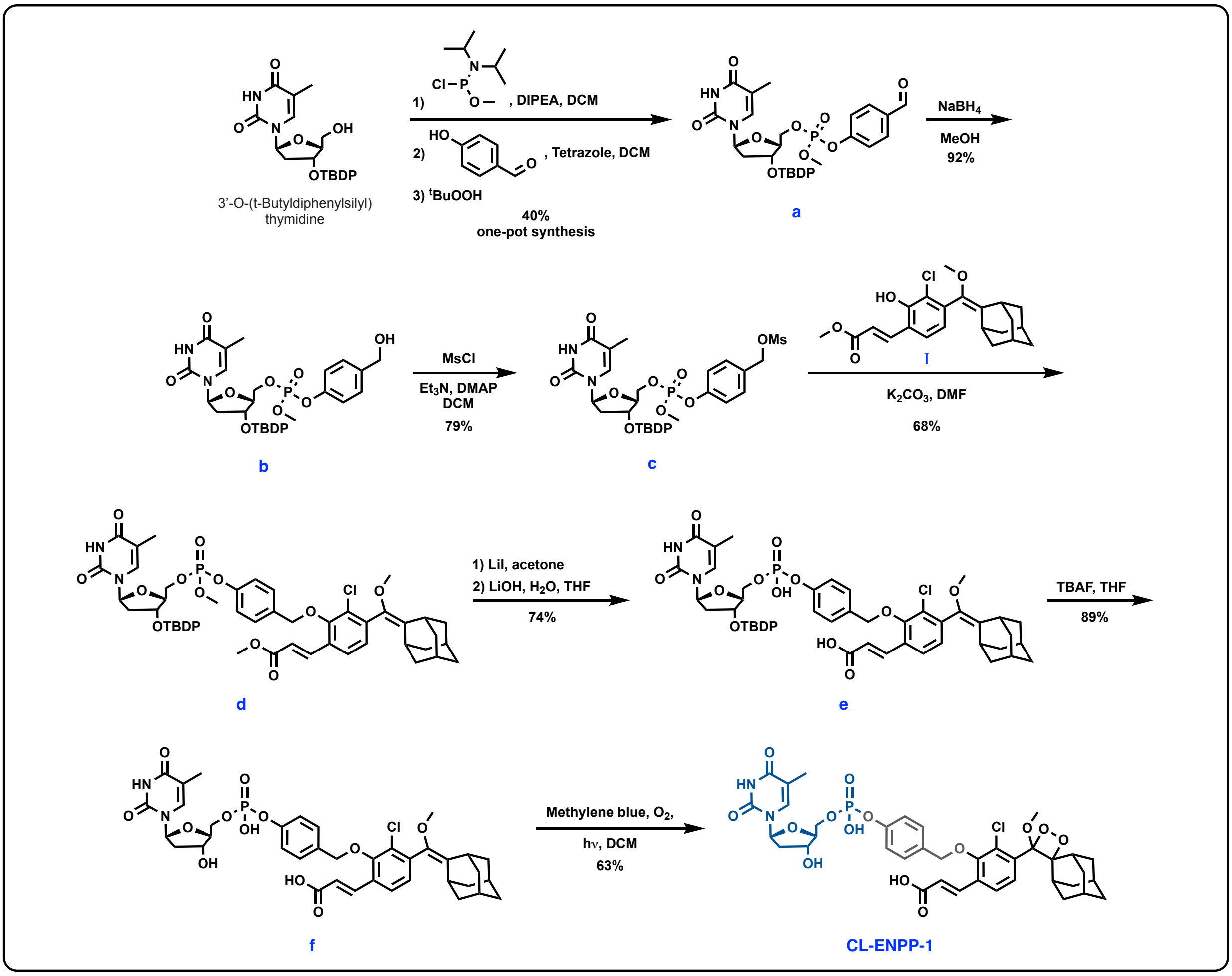

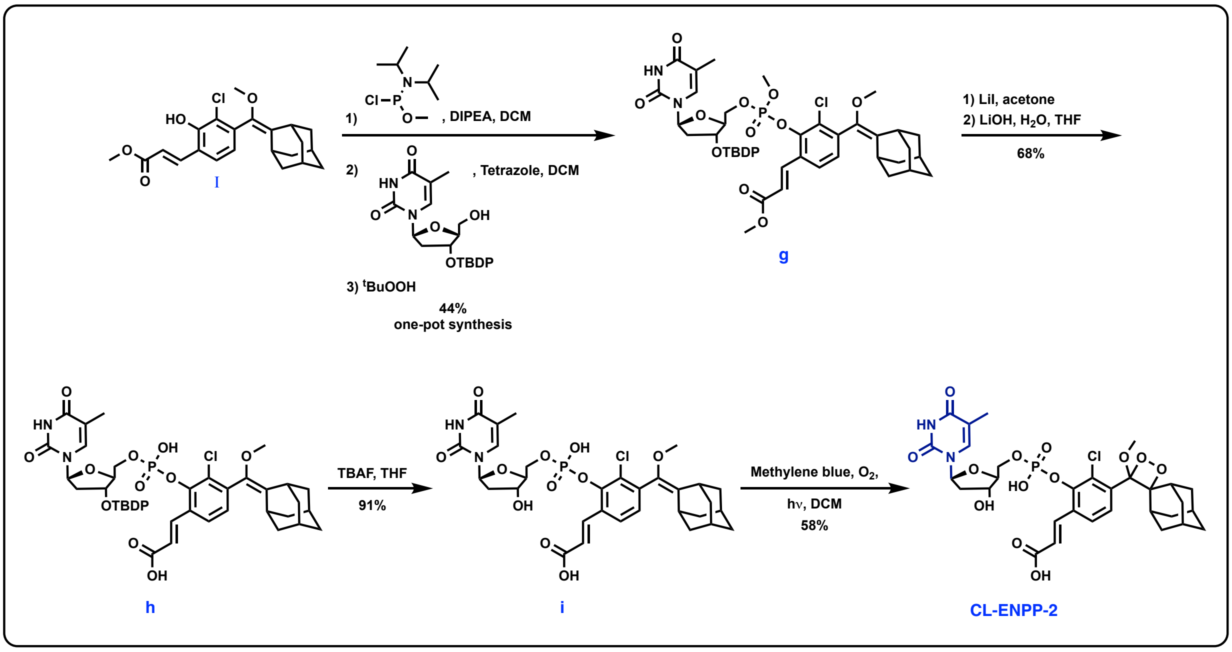

Synthesis of probe CL-ENPP-1

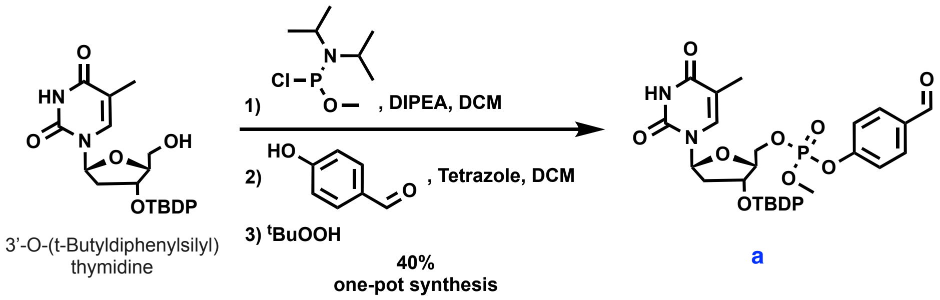

Compound a

3'-O-(t-Butyldiphenylsilyl)thymidine (73 mg, 0.15 mmol, 1 eq.) and DIPEA (78 µL, 0.45 mmol, 3 eq.) were dissolved in 0.5 mL of dry DCM under argon atmosphere and cooled to 0°C. *N,N*-Diisopropylmethylphosphonamidic chloride (60 µL, 0.3 mmol, 2 eq.) dissolved in 0.5 mL of dry DCM was added dropwise and the reaction was monitored by TLC (EtOAc: Hex 7:3). Upon full conversion of 3'-O-(t-Butyldiphenylsilyl)thymidine to the phosphoramidite intermediate, 4-hydroxybenzaldehyde (26 mg, 0.21 mmol, 1.4 eq.) dissolved in 0.5 mL of dry DCM was added to the reaction mixture followed by the addition of tetrazole solution in 0.45M in ACN (0.5 mL, 0.45 mmol, 3 eq.). The reaction was monitored by TLC (EtOAc: Hex 7:3). Upon full conversion of the phosphoramidite intermediate to the phosphite intermediate, ^{t}BuOOH solution in decane 5-6M (60 µL, 0.21 mmol, 2 eq.) was added and the reaction was stirred at room temperature overnight. Upon completion, the reaction mixture was diluted with EtOAc (100 mL) and washed with $NaHCO_3$ (50 mL) and brine (50 mL). The organic layer was separated, dried over Na_2SO_4, and filtered, and the solvent was evaporated under reduced pressure. The crude product was purified by column chromatography on silica gel (EtOAc: Hex 7:3) to afford **compound a** (41 mg, 40% yield) in the form of an off-white solid. MS (ES+): m/z calc. for $C_{34}H_{39}N_2O_9PSi$: 678.75; found:679.8 $[M+H]^+$. **^{1}H NMR** (400 MHz, $CDCl_3$) δ 9.93 (d, *J* = 7.5 Hz, 1H), 9.03 (s, 1H), 7.81 (dd, *J* = 12.7, 8.5 Hz, 2H), 7.69 – 7.55 (m, *J* = 6.2, 4.8 Hz, 4H), 7.50 – 7.32 (m, 6H), 7.31 – 7.13 (m, 3H), 6.44 (dd, *J* = 13.7, 6.4 Hz, 1H), 4.37 (s, 1H), 4.10 – 3.95 (m, 2H), 3.78 (dd, *J* = 11.5, 5.3 Hz, 3H), 3.73 – 3.61 (m, 1H), 2.41 – 2.23 (m, 1H), 1.90 – 1.70 (m, 4H), 1.07 (s, 9H). **^{13}C NMR** (101 MHz, $CDCl_3$) δ 190.65, 163.88, 154.83, 150.41, 135.78, 135.42, 133.74, 133.06, 132.72, 131.85, 131.76, 130.40, 128.17, 120.52, 111.57, 111.44, 85.31, 85.05, 55.38, 40.65, 26.94, 19.10, 12.44. **^{31}P NMR** (162 MHz, $CDCl_3$) δ -6.03.

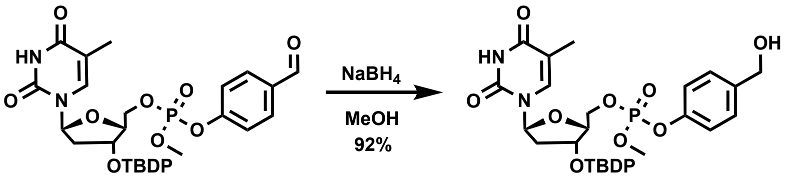

a b

Compound b

Compound **a** (18 mg, 0.02 mmol, 1 eq.) was dissolved in 1 mL of MeOH and cooled to 0°C. $NaBH_4$ (2 mg, 0.04 mmol, 2 eq.) was added to the reaction mixture and the reaction was allowed to warm up to room temperature. The reaction mixture was stirred at room temperature and monitored by TLC (EtOAc: Hex 6:4). Upon completion, the reaction mixture was diluted with EtOAc (100 mL) and washed with NH_4Cl (50 mL) and brine (50 mL). The organic layer was separated, dried over Na_2SO_4, and filtered and the solvent was evaporated under reduced pressure. The crude product was purified by column chromatography on silica gel (EtOAc: Hex 6:4) to afford **compound b** (16.6 mg, 92% yield) in the form of yellow oil. MS (ES+): m/z calc. for $C_{34}H_{41}N_2O_9PSi$: 680.77; found:681.8 $[M+H]^+$. **^{1}H NMR** (400 MHz, $CDCl_3$) δ 8.77 – 8.55 (m, 1H), 7.69 – 7.56 (m, 4H), 7.43 (dd, *J* = 18.1, 7.0 Hz, 6H), 7.29 – 7.18 (m, 3H), 7.02 (d, *J* = 8.2 Hz, 2H), 6.49 – 6.34 (m, 1H), 4.61 (d, *J* = 10.4 Hz, 2H), 4.39 (s, 1H), 4.05 (s, 2H), 3.70 (ddd, *J* = 11.2, 9.6, 6.6 Hz, 4H), 2.42 – 2.22 (m, 1H), 1.91 – 1.78 (m, 2H), 1.72 (d, *J* = 24.1 Hz, 3H), 1.08 (s, 9H). **^{13}C NMR** (101 MHz, $CDCl_3$) δ 164.02, 163.87, 150.45, 150.34, 149.62, 138.56, 135.82, 135.63, 135.53, 133.12, 132.85, 130.37, 128.52, 128.30, 128.16, 120.15, 119.93, 111.43, 111.33, 85.52, 85.27, 84.89, 73.29, 67.55, 64.47, 55.04, 40.82, 40.39, 26.97, 19.12, 12.34. **^{31}P NMR** (162 MHz, $CDCl_3$) δ -5.39.

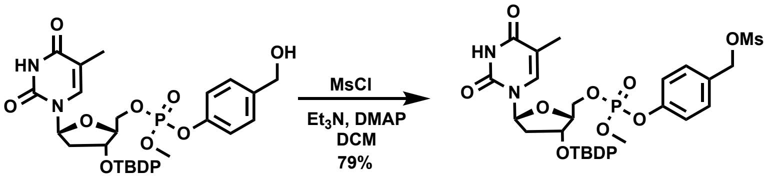

b c

Compound c

Compound **b** (45 mg, 0.07 mmol, 1 eq.), Et_3N (13 μL, 0.09 mmol, 1.3 eq.), and DMAP (2 mg, 0.01 mmol, 0.2 eq.) were dissolved in 1 mL of dry DCM and cooled to 0°C. MsCl (13 μL mg,

0.08 mmol, 1.2 eq.) dissolved in 1 mL of dry DCM was added dropwise and the reaction was allowed to warm up to room temperature. The reaction mixture was monitored by TLC (EtOAc: Hex 7:3). Upon completion, the reaction mixture was diluted with EtOAc (100 mL) and washed with NH_4Cl (50 mL) and brine (50 mL). The organic layer was separated, dried over Na_2SO_4, and filtered and the solvent was evaporated under reduced pressure. The crude product was purified by column chromatography on silica gel (EtOAc: Hex 6:4) to afford **compound b** (39.6 mg, 79% yield) in the form of yellow oil. MS (ES+): m/z calc. for $C_{35}H_{43}N_2O_{11}PSSi$: 758.85; found:781.9 $[M+Na]^+$. **^{1}H NMR** (400 MHz, $CDCl_3$) δ 8.58 (d, *J* = 9.4 Hz, 1H), 7.66 – 7.56 (m, 4H), 7.49 – 7.23 (m, 9H), 7.13 – 7.01 (m, 2H), 6.48 – 6.40 (m, 1H), 5.17 (d, *J* = 8.8 Hz, 2H), 4.39 (s, 1H), 4.05 (s, 2H), 3.78 – 3.70 (m, 3H), 3.64 (d, *J* = 5.5 Hz, 1H), 3.30 (d, *J* = 3.2 Hz, 1H), 2.96 (t, *J* = 9.9 Hz, 3H), 2.32 (t, *J* = 13.6 Hz, 1H), 1.80 (s, 3H), 1.08 (s, 9H). **^{13}C NMR** (101 MHz, $CDCl_3$) δ 163.61, 150.34, 135.80, 135.38, 133.10, 132.80, 130.65, 130.50, 130.38, 128.17, 120.43, 111.49, 85.36, 84.95, 73.18, 70.28, 67.56, 60.54, 55.18, 52.78, 40.65, 38.41, 29.82, 26.96, 19.12, 12.42. **^{31}P NMR** (162 MHz, $CDCl_3$) δ -5.54.

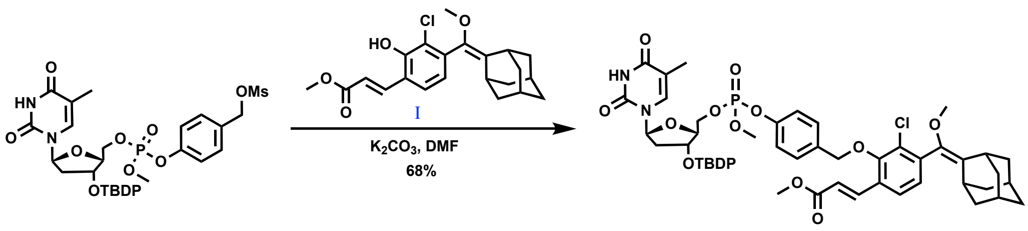

Compound d

Compound **c** (20 mg, 0.27 mmol, 1 eq.) was dissolved in dry DMF (1 mL). K_2CO_3 (7 mg, 0.52 mmol, 2 eq.) was added, and the solution was stirred for 10 minutes at room temperature before compound **I**[1] (10 mg, 0.27 mmol, 1 eq.) was added. The reaction mixture was stirred for 1 hour and monitored by TLC (EtOAc: Hex 7:3). After completion, the reaction mixture was diluted with EtOAc and washed with saturated NH_4Cl. The organic layer was separated, washed with brine, dried over Na_2SO_4, and evaporated under reduced pressure. The crude product was purified by column chromatography (EtOAc: Hex 7:3) to afford **compound d** in the form of a white solid (192 mg, 0.18 mmol, 68%). MS (ES+): m/z calc. for $C_{56}H_{64}ClN_2O_{12}PSi$: 1051.64; found:1074.3 $[M+Na]^+$. **^{1}H NMR** (400 MHz, $CDCl_3$) δ 8.50 (d, *J* = 28.0 Hz, 1H), 7.97 (d, *J* = 16.2 Hz, 1H), 7.63 (d, *J* = 5.2 Hz, 4H), 7.51 – 7.38 (m, 9H), 7.33 (d, *J* = 16.2 Hz, 1H), 7.10 (dd,

J = 15.0, 8.1 Hz, 3H), 6.49 (dt, J = 6.4, 3.2 Hz, 2H), 4.94 (d, J = 7.6 Hz, 2H), 4.41 (s, 1H), 4.06 – 4.00 (m, 1H), 3.82 (s, 3H), 3.65 (s, 2H), 3.35 (d, J = 1.6 Hz, 3H), 3.32 – 3.29 (m, 3H), 2.38 – 2.29 (m, 2H), 2.11 – 1.57 (m, 17H), 1.10 (s, 9H). **^{13}C NMR** (101 MHz, $CDCl_3$) δ 163.44, 150.23, 138.71, 138.30, 135.69, 135.27, 133.49, 132.98, 132.70, 130.24, 130.03, 128.04, 125.15, 119.93, 115.88, 111.42, 85.35, 84.84, 75.11, 73.20, 67.47, 57.32, 55.03, 52.66, 51.88, 40.60, 39.05, 38.66, 37.04, 32.96, 31.93, 31.63, 29.70, 29.36, 28.19, 26.84, 22.70, 18.99, 14.13, 12.30. **^{31}P NMR** (162 MHz, $CDCl_3$) δ -5.43.

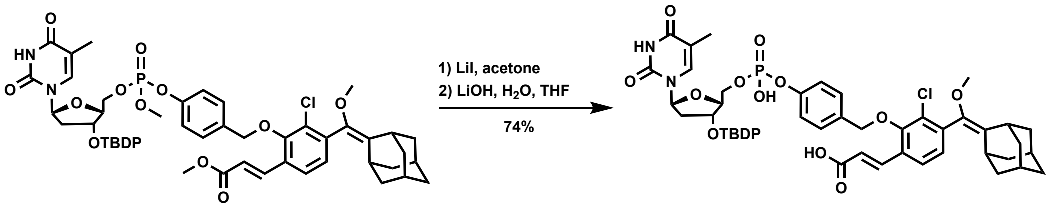

d

e

Compound e

Compound **d** (15 mg, 0.01 mmol, 1 eq.) was dissolved in acetone (0.5 mL). LiI (4 mg, 0.02 mmol, 2 eq.) was added, and the solution was stirred at room temperature for 30 minutes. The reaction was monitored by RP-HPLC (70-100% ACN, ammonium carbonate buffer [30 mM]). Upon completion, the acetone was evaporated, and the reaction mixture was dissolved in THF:H_2O mixture (4:1). LiOH (4 mg, 0.05 mmol, 10 eq.) was added and the reaction was heated to 50°C. The reaction was monitored by RP-HPLC (70-100% ACN, ammonium carbonate buffer [30 mM]). After completion, the reaction mixture was concentrated by evaporation under reduced pressure. The crude product was purified by preparative RP-HPLC (70-100% ACN, ammonium carbonate buffer [30 mM]; flow rate: 20 mL/min; retention time: 4 min) to afford **compound e** in the form of a white solid (11 mg, 74% yield). MS (ES+): m/z calc. for $C_{54}H_{60}ClN_2O_{12}PSi$: 1023.58; found:1022.3 $[M-H]^-$. **^{1}H NMR** (400 MHz, DMSO) δ 7.70 (s, 2H), 7.57 (t, J = 6.8 Hz, 4H), 7.50 (d, J = 7.5 Hz, 1H), 7.35 (dd, J = 14.0, 6.8 Hz, 6H), 7.20 (d, J = 7.5 Hz, 2H), 7.06 (d, J = 7.7 Hz, 1H), 7.01 (d, J = 7.8 Hz, 2H), 6.52 – 6.44 (m, 1H), 6.40 (d, J = 15.3 Hz, 1H), 4.87 (s, 2H), 4.45 (s, 1H), 4.04 (s, 1H), 3.94 (d, J = 11.0 Hz, 1H), 3.54 (d, J = 11.0 Hz, 1H), 3.36 – 3.27 (m, 3H), 3.24 (s, 1H), 2.19 – 1.66 (m, 18H), 1.25 (s, 1H), 1.04 (s, 9H). **^{13}C NMR** (101 MHz, DMSO) δ 165.12, 153.49, 153.03, 151.27, 139.86, 137.09, 136.66, 135.59, 133.16, 133.00, 131.54, 131.20, 130.09, 129.44, 127.87, 127.81, 127.68, 124.87, 119.75, 110.95, 86.71, 85.02,

78.00, 75.61, 74.90, 65.73, 56.16, 40.16, 38.89, 38.33, 36.89, 33.13, 29.80, 28.57, 28.43, 26.14, 18.49, 11.35. **^{31}P NMR** (162 MHz, DMSO) δ -6.66.

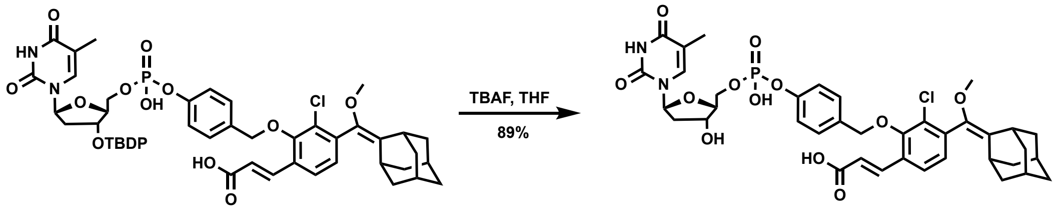

e f

Compound f

Compound **e** (3 mg, 0.002 mmol, 1 eq.) was dissolved in THF (0.5 mL) under an argon atmosphere. TBAF (1.0 M in THF, 0.02 mmol, 10 eq.) was added dropwise, and the solution was stirred for 5 minutes. The reaction was monitored by RP-HPLC (30-100% ACN, ammonium carbonate buffer [30 mM]). Upon completion, the solvent was concentrated under reduced pressure, and the product was purified by preparative RP-HPLC (30-100% ACN, ammonium carbonate buffer [30 mM]; flow rate: 20 mL/min; retention time: 12 min). **Compound f** was obtained as a white solid (2.0 mg, 89% yield). MS (ES-): m/z calc. for $C_{38}H_{42}ClN_2O_{12}P$: 784.22; found:783.21 [M-H]$^-$. **^{1}H NMR** (400 MHz, MeOD) δ 7.79 (s, 1H), 7.71 (s, 1H), 7.49 (d, *J* = 7.8 Hz, 1H), 7.37 – 7.23 (m, 3H), 7.18 (d, *J* = 7.9 Hz, 2H), 7.05 (d, *J* = 7.9 Hz, 1H), 6.33 (t, *J* = 6.9 Hz, 2H), 4.96 (d, *J* = 3.5 Hz, 2H), 4.52 (s, 1H), 4.45 (s, 1H), 4.17 (s, 1H), 4.04 (s, 1H), 3.24 (s, 3H), 2.23 (d, *J* = 5.2 Hz, 3H), 2.07 (s, 1H), 2.02 – 1.57 (m, 17H). **^{13}C NMR** (101 MHz, MeOD) δ 165.17, 153.58, 151.17, 139.81, 136.67, 134.73, 131.82, 131.37, 130.23, 129.38, 127.66, 127.35, 124.71, 120.05, 110.88, 86.06, 85.97, 84.90, 71.35, 65.68, 65.13, 56.39, 39.70, 38.96, 38.85, 38.50, 38.39, 36.95, 33.10, 29.80, 28.54, 28.39, 26.03, 23.57, 19.51, 12.86, 11.52. **^{31}P NMR** (162 MHz, MeOD) δ -6.48.

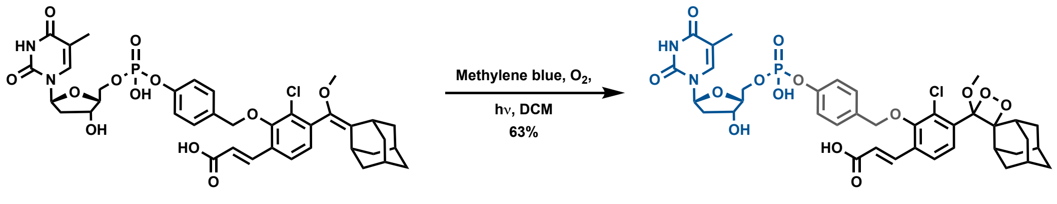

f

CL-ENPP-1

Probe CL-ENPP-1

Compound **f** (2 mg, 0.002 mmol, 1 eq.) and a catalytic amount of methylene blue (~1 mg) were dissolved in 10 mL of DCM. Oxygen was bubbled through the solution while irradiating with yellow light., and the reaction was monitored by RP-HPLC (30-100% ACN, ammonium carbonate buffer [30 mM]). Upon completion, the solvent was concentrated under reduced pressure, and the product was purified by preparative RP-HPLC (30-100% ACN, ammonium carbonate buffer [30 mM]; flow rate: 20 mL/min; retention time: 12 min). **Probe CL-ENPP-1** was obtained as a white solid (1.3 mg, 63% yield). MS (ES+): m/z calc. for $C_{38}H_{42}ClN_2O_{14}P$: 816.21; found:815.15 $[M-H]^-$. **^{1}H NMR** (400 MHz, 5% MeOD in DMSO) δ 7.82 – 7.57 (m, 3H), 7.15 (t, *J* = 19.3 Hz, 5H), 6.36 (s, 1H), 6.26 – 6.12 (m, 1H), 4.83 (d, *J* = 10.3 Hz, 2H), 4.28 (s, 1H), 3.98 (s, 1H), 3.91 (s, 1H), 3.46 (s, 1H), 3.22 – 3.05 (m, 3H), 2.89 (s, 1H), 2.27 (s, 1H), 2.18 – 1.99 (m, 2H), 1.93 (s, 1H), 1.81 – 1.16 (m, 16H). **^{13}C NMR** (101 MHz, 5% MeOD in DMSO) δ 164.25, 162.76, 153.81, 151.26, 151.01, 144.13, 140.23, 136.64, 130.54, 128.70, 127.17, 120.21, 111.74, 110.23, 95.88, 86.22, 84.26, 76.23, 71.45, 65.59, 50.40, 49.88, 36.30, 33.77, 33.53, 32.17, 31.54, 31.29, 25.97, 25.66, 12.54. **^{31}P NMR** (162 MHz, 5% MeOD in DMSO) δ -6.30.

Synthesis of probe CL-ENPP-2

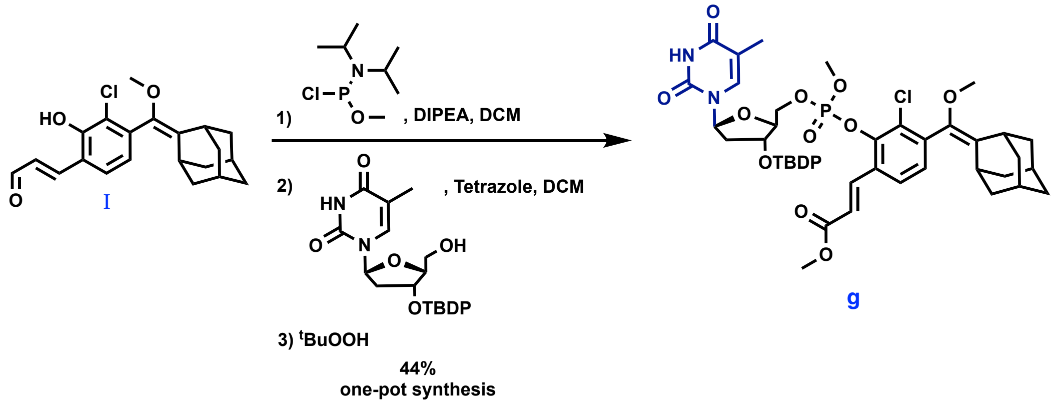

Compound g

Compound **I**[1] (140 mg, 0.36 mmol, 1 eq.) and DIPEA (78 μL, 0.45 mmol, 3 eq.) were dissolved in 0.5 mL of dry DCM under argon atmosphere and cooled to 0°C. *N,N*-Diisopropylmethylphosphonamidic chloride (76 μL, 0.3 mmol, 1.1 eq.) dissolved in 0.5 mL of dry DCM was added dropwise and the reaction was monitored by TLC (EtOAc: Hex 3:7). Upon full conversion of Compound **I** to the phosphoramidite intermediate, 3'-O-(t-Butyldiphenylsilyl)thymidine (170 mg, 0.36 mmol, 1 eq.) dissolved in 0.5 mL of dry DCM was added to the reaction mixture followed by the addition of tetrazole solution in 0.45M in ACN (800 μL, 0.72 mmol, 2 eq.). The reaction was monitored by TLC (EtOAc: Hex 3:7). Upon full conversion of the phosphoramidite intermediate to the phosphite intermediate, ^{t}BuOOH solution in decane 5-6M (70 μL, 0.36 mmol, 1 eq.) was added and the reaction was stirred at room temperature overnight. Upon completion, the reaction mixture was diluted with EtOAc (100 mL) and washed with $NaHCO_3$ (50 mL) and brine (50 mL). The organic layer was separated, dried over Na_2SO_4, and filtered, and the solvent was evaporated under reduced pressure. The crude product was purified by column chromatography on silica gel (EtOAc: Hex 7:3) to afford **compound g** (149 mg, 44% yield) in the form of an off-white solid. MS (ES+): m/z calc. for $C_{49}H_{58}ClN_2O_{11}PSi$: 945.51; found:946.2 $[M+H]^+$. **^{1}H NMR** (400 MHz, $CDCl_3$) δ 8.52 (s, 1H), 7.92 (dd, *J* = 16.0, 3.9 Hz, 1H), 7.69 – 7.58 (m, 4H), 7.52 – 7.33 (m, 8H), 7.14 (d, *J* = 8.0 Hz, 1H), 6.53 – 6.35 (m, 2H), 4.46 (s, 1H), 4.23 – 4.14 (m, 2H), 3.86 – 3.67 (m, 7H), 3.33 – 3.20 (m, 4H), 2.33 (dd, *J* = 13.3, 3.4 Hz, 1H), 2.16 (d, *J* = 2.6 Hz, 2H), 1.99 – 1.60 (m, 13H), 1.08 (s, 9H). **^{13}C NMR** (101 MHz, $CDCl_3$) δ 166.76, 163.73, 150.33, 145.72, 139.13, 138.56, 137.75, 135.80, 135.64, 133.10, 132.91, 130.35, 129.15, 128.53, 128.38, 128.15, 124.88, 121.12, 121.02,

111.48, 111.36, 85.52, 85.11, 85.02, 73.48, 68.26, 68.02, 60.55, 57.50, 55.74, 52.03, 40.77, 39.29, 39.14, 38.73, 37.08, 33.05, 31.06, 29.86, 28.40, 28.26, 26.98, 19.14, 14.32, 12.35, 12.28. **^{31}P NMR** (162 MHz, $CDCl_3$) δ -5.49, -5.78.

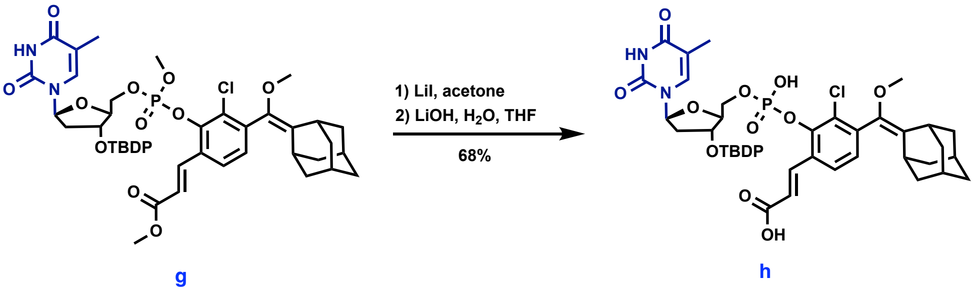

Compound h

Compound **g** (70 mg, 0.07 mmol, 1 eq.) was dissolved in acetone (1 mL). LiI (20 mg, 0.14 mmol, 2 eq.) was added, and the solution was stirred at room temperature for 30 minutes. The reaction was monitored by RP-HPLC (70-100% ACN, ammonium carbonate buffer [30 mM]). Upon completion, the acetone was evaporated, and the reaction mixture was dissolved in THF:H_2O mixture (4:1). LiOH (18 mg, 0.7 mmol, 10 eq.) was added and the reaction was heated to 50°C. The reaction was monitored by RP-HPLC (70-1000% ACN, ammonium carbonate buffer [30 mM]). After completion, the reaction mixture was concentrated by evaporation under reduced pressure. The crude product was purified by preparative RP-HPLC (90-100% ACN, ammonium carbonate buffer [30 mM]; flow rate: 20 mL/min; retention time: 6 min) to afford **compound h** in the form of a white solid (43 mg, 68% yield). MS (ES+): m/z calc. for $C_{47}H_{54}ClN_2O_{11}PSi$: 916.29; found:915.3 $[M-H]^-$. **^{1}H NMR** (400 MHz, DMSO) δ 8.02 (d, *J* = 16.3 Hz, 1H), 7.81 (s, 1H), 7.65 – 7.51 (m, 5H), 7.48 – 7.33 (m, 6H), 6.88 (d, *J* = 8.1 Hz, 1H), 6.34 (d, *J* = 15.5 Hz, 2H), 4.52 (s, 1H), 4.02 (s, 1H), 3.89 (s, 1H), 3.66 (s, 1H), 3.13 (s, 3H), 2.07 – 1.78 (m, 6H), 1.78 – 1.52 (m, 10H), 1.01 (s, 9H). **^{13}C NMR** (101 MHz, DMSO) δ 164.36, 151.21, 140.51, 136.90, 135.78, 133.48, 130.62, 129.96, 128.58, 126.13, 124.90, 110.61, 87.08, 84.40, 75.56, 65.82, 56.93, 39.02, 38.56, 37.11, 32.85, 29.55, 28.24, 27.32, 19.19, 12.49. **^{31}P NMR** (162 MHz, DMSO) δ -5.74.

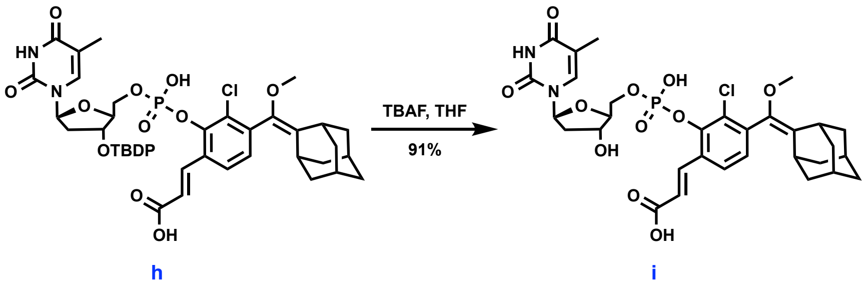

Compound i

Compound **h** (10 mg, 0.01 mmol, 1 eq.) was dissolved in THF (0.5 mL) under an argon atmosphere. TBAF (1.0 M in THF, 0.1 mmol, 10 eq.) was added dropwise, and the solution was stirred for 5 minutes. The reaction was monitored by RP-HPLC (30-100% ACN, ammonium carbonate buffer [30 mM]). Upon completion, the solvent was concentrated under reduced pressure, and the product was purified by preparative RP-HPLC (30-100% ACN, ammonium carbonate buffer [30 mM]; flow rate: 20 mL/min; retention time: 3.5 min). **Compound i** was obtained as a white solid (6.7 mg, 91% yield). MS (ES-): m/z calc. for $C_{31}H_{36}ClN_2O_{11}P$: 678.17; found:677.17 $[M-H]^-$. **^{1}H NMR** (400 MHz, MeOD) δ 8.18 (d, *J* = 15.6 Hz, 1H), 7.80 (s, 1H), 7.61 (d, *J* = 7.8 Hz, 1H), 7.06 (d, *J* = 7.9 Hz, 1H), 6.47 (d, *J* = 15.8 Hz, 1H), 6.33 (t, *J* = 6.7 Hz, 1H), 4.62 – 4.47 (m, 2H), 4.44 – 4.20 (m, 2H), 4.08 (s, 1H), 3.23 (s, 1H), 2.23 (s, 2H), 2.16 – 2.02 (m, 1H), 1.99 – 1.60 (m, 13H), 1.45 – 1.19 (m, 4H). **^{13}C NMR** (101 MHz, MeOD) δ 165.19, 151.14, 140.08, 136.82, 136.44, 136.29, 131.50, 130.21, 128.29, 127.26, 123.88, 110.62, 86.23, 86.15, 84.92, 71.50, 66.20, 66.13, 56.14, 39.67, 38.84, 38.40, 36.90, 32.99, 29.79, 28.58, 28.42, 11.28. **^{31}P NMR** (162 MHz, MeOD) δ -6.99.

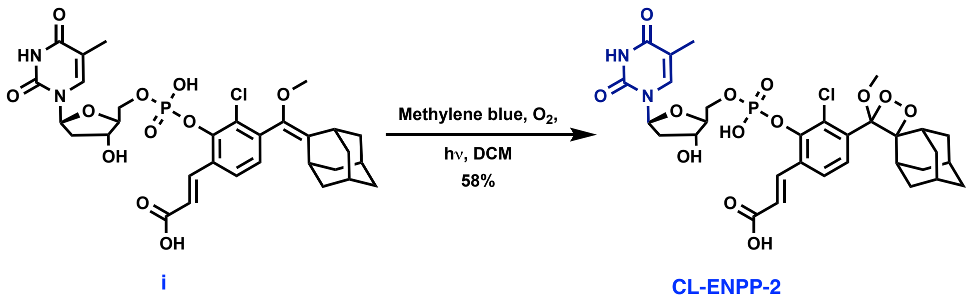

Probe CL-ENPP-2

Compound **i** (6.7 mg, 0.01 mmol, 1 eq.) and a catalytic amount of methylene blue (~1 mg) were dissolved in 10 mL of DCM. Oxygen was bubbled through the solution while irradiating

with yellow light., and the reaction was monitored by RP-HPLC (30-100% ACN, ammonium carbonate buffer [30 mM]). Upon completion, the solvent was concentrated under reduced pressure, and the product was purified by preparative RP-HPLC (30-100% ACN, ammonium carbonate buffer [30 mM]; flow rate: 20 mL/min; retention time: 3.5 min). **Probe CL-ENPP-2** was obtained as a white solid (4.1 mg, 58% yield). MS (ES+): m/z calc. for $C_{31}H_{36}ClN_2O_{13}P$: 710.16; found:709.15 $[M-H]^-$. **^{1}H NMR** (400 MHz, 5% MeOD in DMSO) δ 8.02 (d, *J* = 14.2 Hz, 1H), 7.82 – 7.57 (m, 3H), 6.40 (d, *J* = 16.1 Hz, 1H), 6.26 – 6.14 (m, 1H), 4.37 (s, 1H), 3.92 (s, 1H), 3.38 (s, 3H), 2.86 (s, 1H), 2.30 – 2.11 (m, 3H), 1.99 (m, 3H), 1.77 – 1.36 (m, 15H).**^{31}P NMR** (162 MHz, 5% MeOD in DMSO) δ -6.68.

Experimental protocols

Chemiluminescent measurements of probes CL-ENPP-1 and CL-ENP-2

All stock solutions were prepared in DMSO at a final concentration of 10 mM. Measurements were recorded by Spectramax iD3 with integration time parameters set at 140 msec. The measurements were conducted in a white 96-well Corning™ plate, in a final well volume of 100 μL, 1% DMSO unless otherwise mentioned. Recombinant Human ENPP-1 (R&D systems, Catalog number: 6136-EN) [0.1 μg/mL] was added to each well and the light emission was recorded immediately. All measurements were repeated in three independent experiments.

ENPP-1 Limit-of-detection measurements

All stock solutions were prepared in DMSO at a final concentration of 10 mM. Measurements were recorded by Spectramax iD3 with integration time parameters set at 140 msec. The measurements were conducted in a white 96-well Corning™ plate, in a final well volume of 100 mL, 3% DMSO. To **CL-ENPP** [30 μM] and **TMP-pNP** [300 μM] in PBS pH 7.4, at 37°C was added a serial dilution of Recombinant Human ENPP-1 [0.1 μg/mL - 1 pg/mL] and the light emission was recorded immediately. All measurements were repeated in three independent experiments.

Evaluation of the selectivity of the probe towards ENPP-1 compared to alkaline phosphatase

A comparison between the activation of **TMP-pNP** [300 μM] and **CL-ENPP** and **CL-ENPP-2** [10 μM] in the presence of standard concentration Recombinant Human ENPP-1 [0.1 μg/mL] and Alkaline phosphatase [0.075U/mL]. The measurements were conducted using the above-mentioned procedure.

Detection of ENPP-1 activity in cancer cells in vitro

Cell culture

MDA-MB-231 cells were cultured in RPMI 1640 Medium supplemented with 10% FBS, 1% streptomycin, 1% penicillin, and 1% L-glutamine. Cells were grown at 37°C; 5% CO_2.

Chemiluminescent probe activation in vitro

For testing the activity of probes **CL-ENPP-2** and **TMP-pNP**, MDA-MB-231 cells were seeded in 96-well clear bottom plates at a concentration of 40,000 cells/well in 100 μL medium. 24 hours after seeding, the cell media were removed, the monolayer of cells was washed twice with PBS and treated with probe **CL-ENPP-2** [10 μM, 0.1% DMSO] or **TMP-pNP** [300 μM, 0.1% DMSO]. The chemiluminescence signal was recorded immediately using a Molecular Devices Spectramax iD3, as noted above.

Supplementary Figures

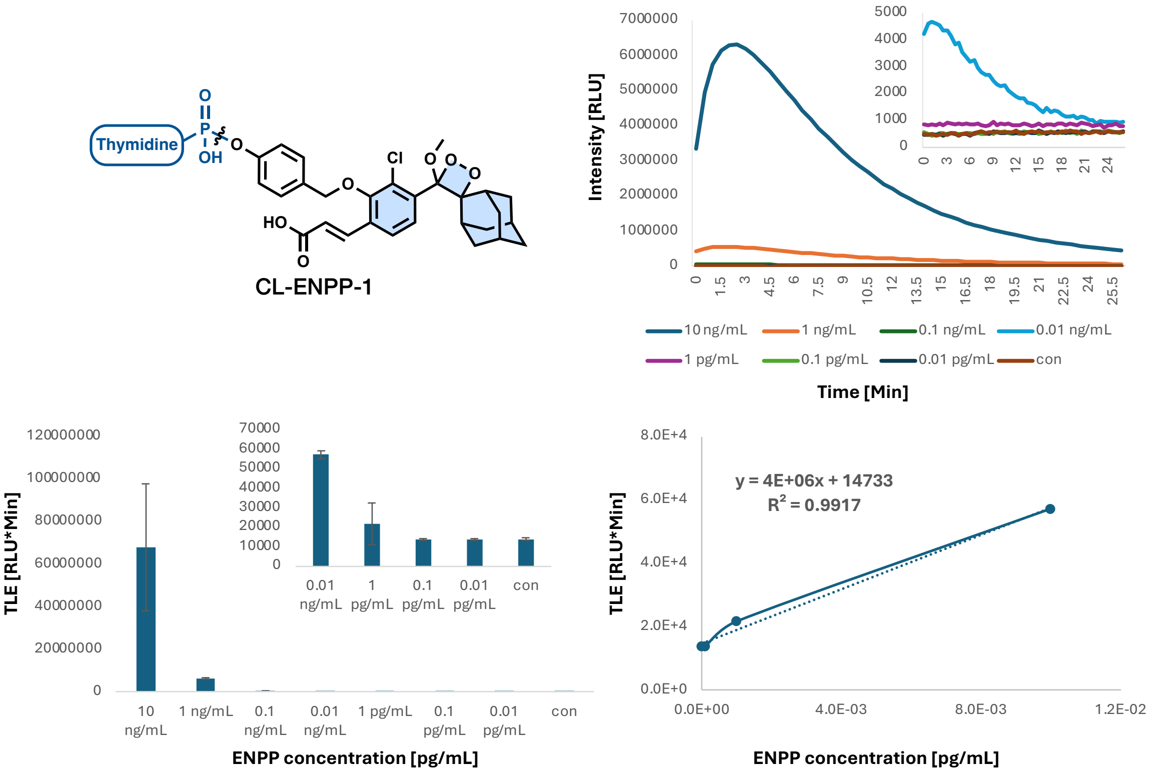

Figure S1. Probe CL-ENPP-1 Limit-of-detection (L.O.D) measurements. (Top left) chemical structure of CL-ENPP-1, (Top right) chemiluminescent kinetic profiles (Bottom left) total light emission, and (Bottom right), Linear calibration curve of **CL-ENPP-1** [30 μM] in PBS pH 7.4, 3% DMSO, 37°C with or without Recombinant Human ENPP-1 [0.01 μg/mL - 1 pg/mL].The limit of detection was determined as 3*(S.D. of the blank) divided by the slope of the linear calibration curve (L.O.D = 3σ/k). Error bars are S.D. from three independent experiments.

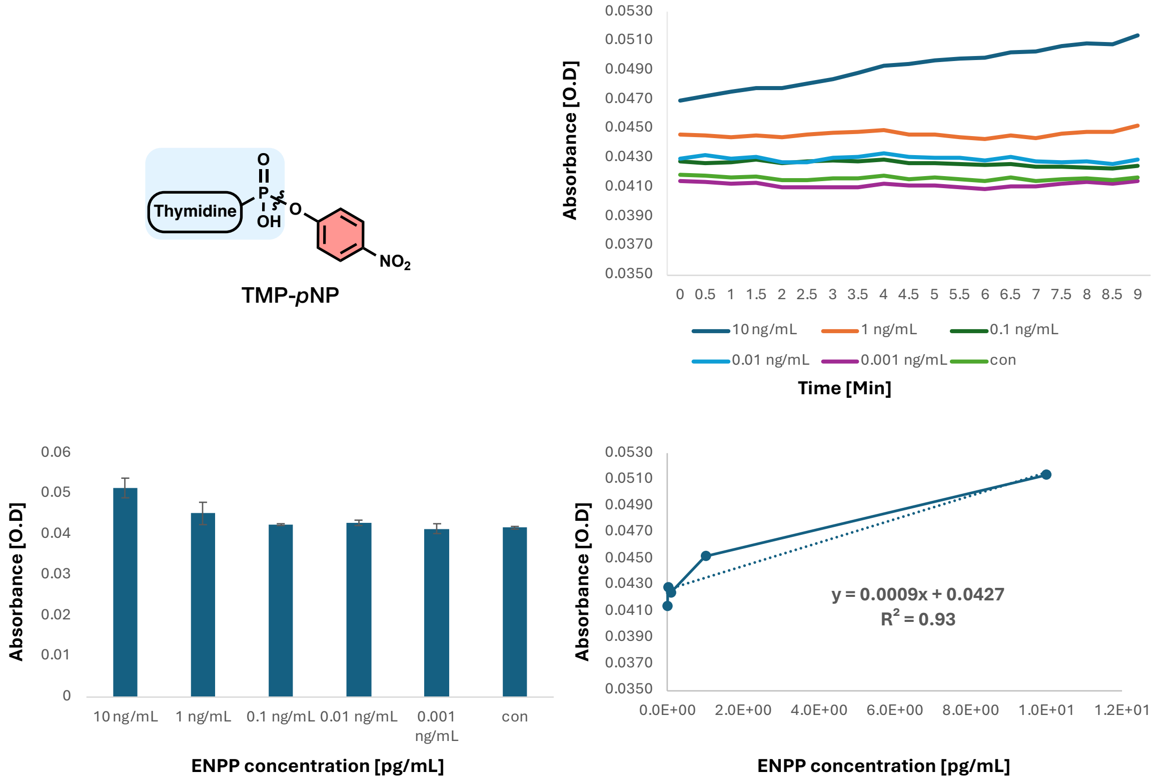

Figure S2. Probe **TMP-pNP** Limit-of-detection (L.O.D) measurements. (Top left) chemical structure of **TMP-pNP**, (Top right) kinetic profile, (Bottom left) end-point absorbance, and (Bottom right) Linear calibration curve of **TMP-pNP** [300 μM] in PBS pH 7.4, 3% DMSO, 37°C with or without Recombinant Human ENPP-1 [0.01 μg/mL - 1 pg/mL]. The limit of detection was determined as 3*(S.D. of the blank) divided by the slope of the linear calibration curve (L.O.D = 3σ/k). Error bars are S.D. from three independent experiments.

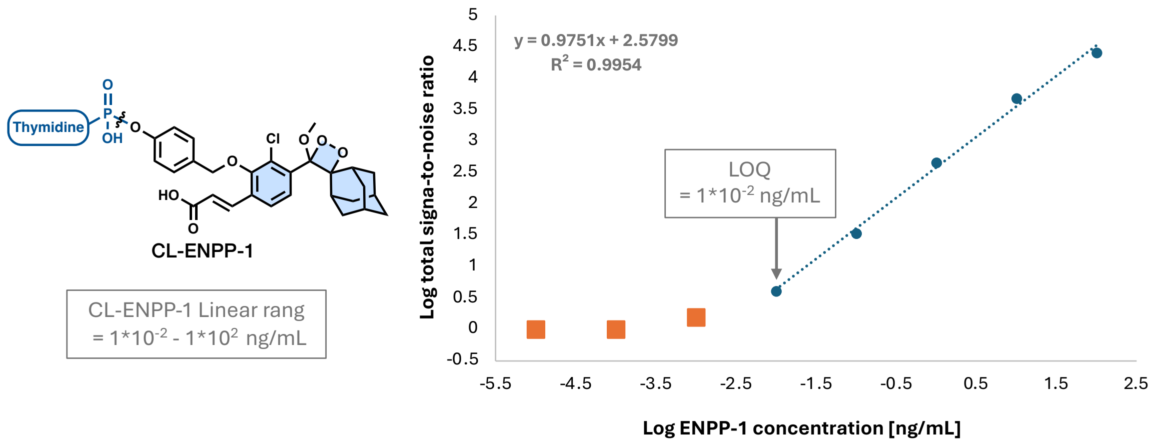

Figure S3. Probe CL-ENPP-1 linear response measurements. (Left) Chemical structure of CL-ENPP-1. (Right) Linear fit of CL-ENPP-1 [10 μM] in PBS (pH 7.4) with 0.1% DMSO at 37°C in the presence of serial dilutions of recombinant human ENPP-1 [0.1 μg/mL – 1 pg/mL]. The lowest point of the linear range (lower limit of quantification, **LOQ**) was defined as the lowest concentration within the linear response range that maintains an $R^2 > 0.99$.

HPLC and Mass Spectra

CL-ENPP-1

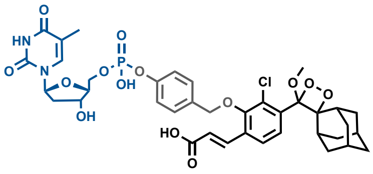

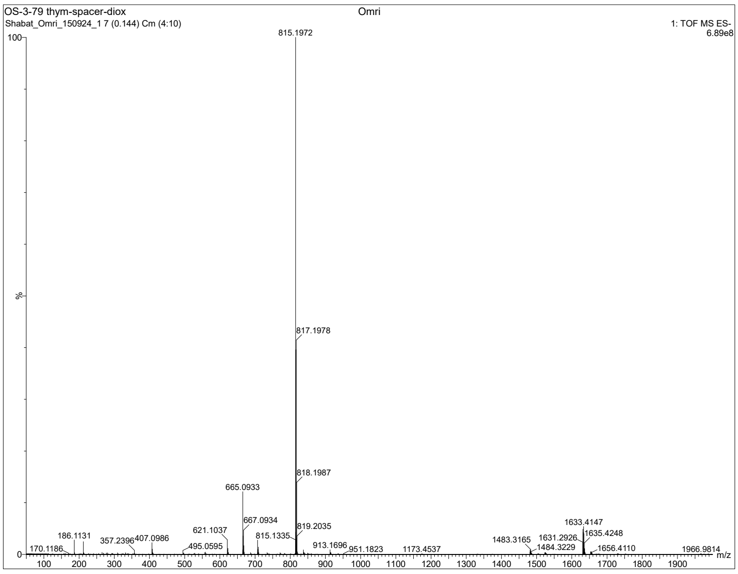

Mass spectra

Chemical Formula: $C_{38}H_{42}ClN_2O_{14}P$

Exact Mass: 816.21

3D HPLC spectra (50-100% ACN in water, 0.1%TFA)

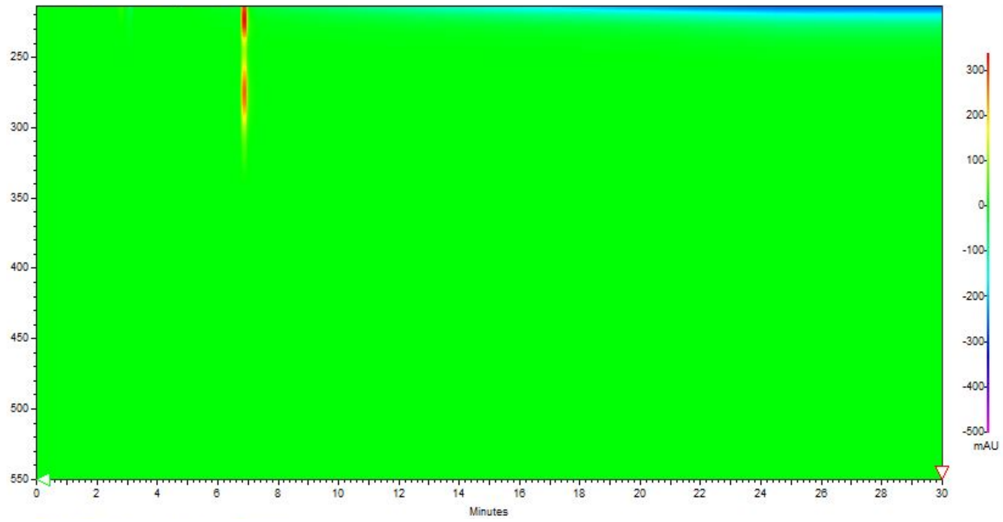

2D HPLC spectra (Absorbance measured at 278 nm)

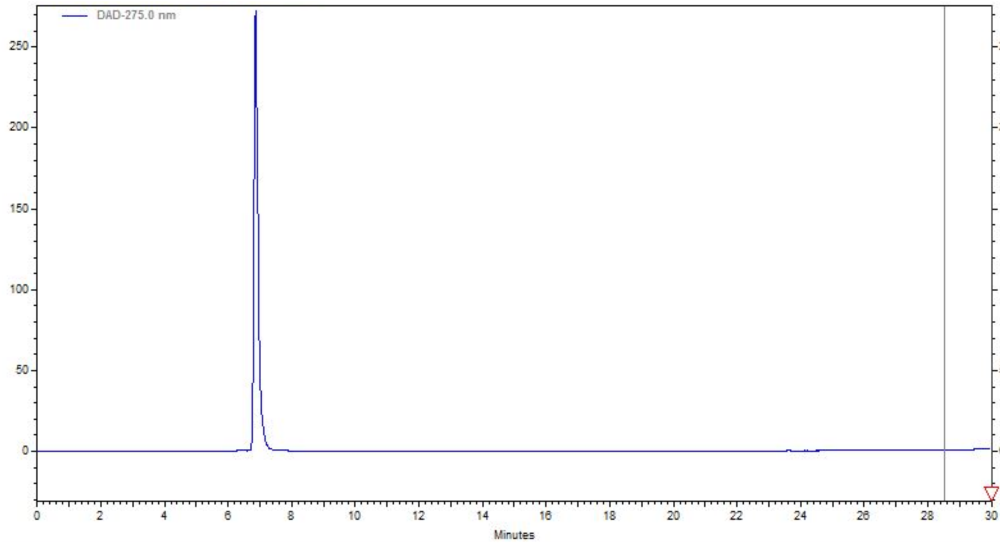

CL-ENPP-2

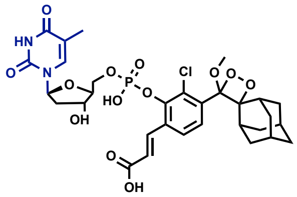

CL-ENPP-2

Chemical Formula: $C_{31}H_{36}ClN_2O_{13}P$
Exact Mass: 710.16

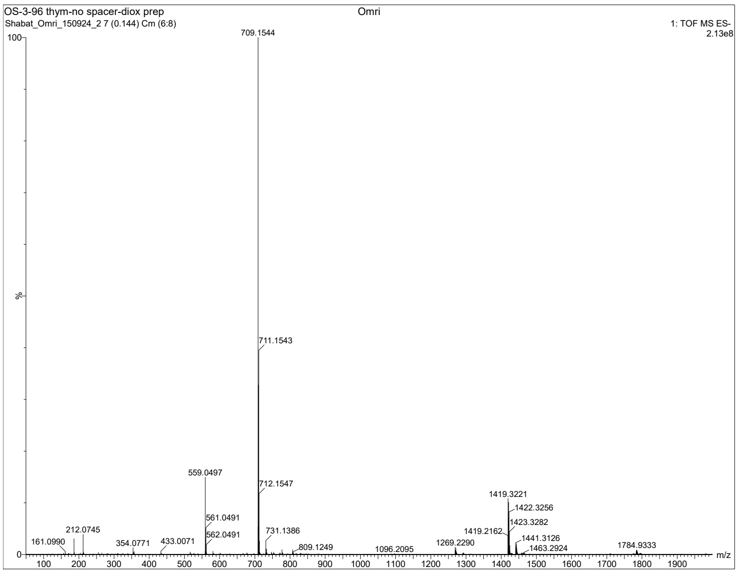

Mass spectra

Chemical Formula: $C_{31}H_{36}ClN_2O_{13}P$

Exact Mass: 710.16

3D HPLC spectra (30-100% ACN in water, 0.1%TFA)

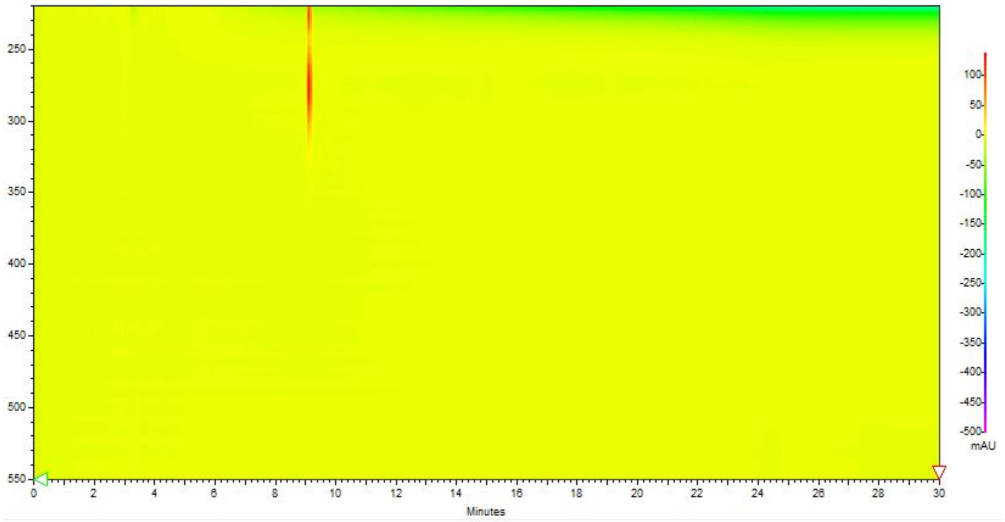

2D HPLC spectra (Absorbance measured at 278 nm)

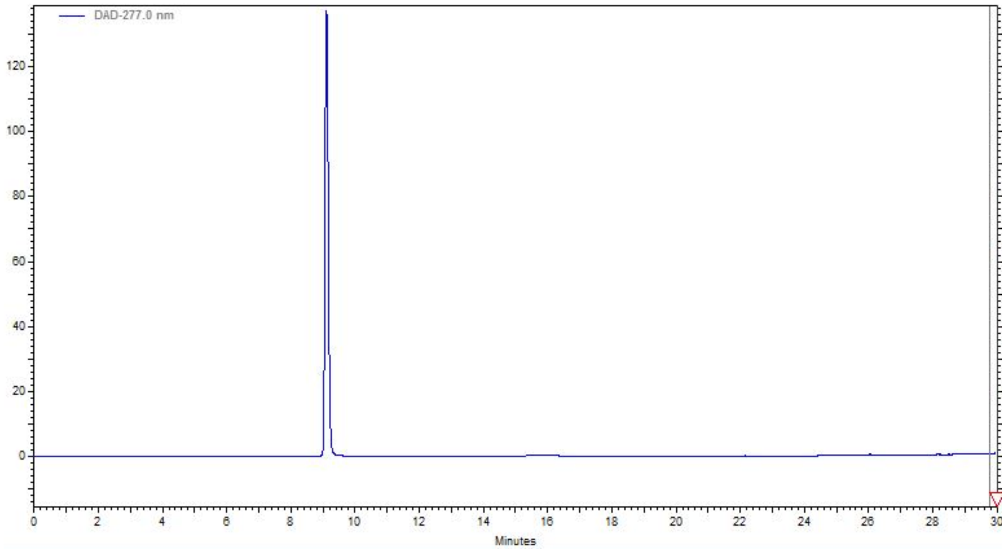

References

(1) Hananya, N.; Reid, J. P.; Green, O.; Sigman, M. S.; Shabat, D. Rapid chemiexcitation of phenoxy-dioxetane luminophores yields ultrasensitive chemiluminescence assays. *Chem. Sci.* **2019**, *10* (5), 1380-1385. DOI: 10.1039/c8sc04280b.
